# Cancer-associated fibroblast-induced lncRNA WARS2-IT1 confers radioresistance of colorectal cancer *via* enhancing HIF-1α stability

**DOI:** 10.1038/s41419-025-08058-1

**Published:** 2025-11-10

**Authors:** Yuanqi Li, Wei Dai, Xiao Zheng, Qi Wang, Jinping Zhang, Xiangyin Kong, Jingting Jiang, You Zhou

**Affiliations:** 1 Tumor Biological Diagnosis and Treatment Center, The Third Affiliated Hospital of Soochow University, Changzhou, China; 2 Jiangsu Engineering Research Center for Tumor Immunotherapy, Changzhou, China; 3 Institute of Cell Therapy, Soochow University, Changzhou, China; 4 Institutes of Biology and Medical Sciences, Soochow University, Suzhou, China; 5 CAS Key Laboratory of Tissue Microenvironment and Tumor, Shanghai Institute of Nutrition and Health, Chinese Academy of Sciences, Shanghai, China

**Keywords:** Cancer microenvironment, Oncogenes

## Abstract

The tumor microenvironment in colorectal cancer (CRC) is marked by a diverse and abundant population of cancer-associated fibroblasts (CAFs), which play a crucial role in radioresistance. Nonetheless, the mechanisms through which CAFs contribute to radioresistance remain unclear. In this study, we demonstrate that CAF^R^, a specific subset of CAFs derived from radioresistant CRC patients, produces higher levels of transforming growth factor-β1 (TGF-β1) compared to CAFs isolated from radiosensitive CRC patients. Through long noncoding RNA (lncRNA) profiling of tumor cells treated with CAF-conditioned medium (CAF-CM), we identify WARS2-IT1 (WARS2 intronic transcript 1), whose expression is directly stimulated by TGF-β1 signaling. This lncRNA serves as a key player in promoting radioresistance and is essential for the TGFβ1-induced radioresistance pathway. Mechanistically, WARS2-IT1 interferes with the interaction between prolyl hydroxylase domain 2 (PHD2) and hypoxia-inducible factor-1α (HIF-1α), preventing the hydroxylation and subsequent degradation of HIF-1α. This process leads to the activation of glycolytic pathways, thereby enhancing radioresistance. Our findings underscore the potential of targeting CAF-driven WARS2-IT1 as a promising strategy to counteract tumor radioresistance in CRC.

## Introduction

Colorectal cancer (CRC) poses a significant global public health challenge, consistently ranking among the most prevalent and lethal malignancies [1, 2]. Despite progress in treatments like surgery, chemotherapy, and targeted therapies, overcoming radioresistance remains a critical barrier to improving outcomes for CRC patients [3–5]. This resistance may arise from multiple elements in the surrounding environment of the tumor, with CAFs being crucial contributors [6]. As the most abundant stromal cell type, CAFs are integral to numerous aspects of tumor biology. These heterogeneous cells, residing in the stroma of solid tumors, including CRC, actively shape the tumor microenvironment by releasing growth factors, cytokines, and extracellular matrix components that regulate tumor growth, invasion, metastasis, and therapeutic responses [7]. Recent studies have underscored their contribution to radiotherapy resistance, complicating treatment outcomes and limiting efficacy [8]. Thus, a deeper understanding of the roles and mechanisms of specific CAF subpopulations is crucial for developing effective targeted therapies.

Chemokines, the small chemotactic cytokines expressed by tumor and stromal cells, play essential roles in orchestrating cell positioning and trafficking [9]. Abnormal expression of chemokines and their associated receptors has been associated with a range of human diseases, including cancers, autoimmune conditions, and inflammatory disorders. Additionally, chemokines facilitate critical cell–cell interactions by recruiting and activating different cell types to tumor sites through autocrine or paracrine mechanisms [10, 11]. Chemokines produced by stromal cells have been shown to be involved in tumor growth, metastasis, and angiogenesis [11]. In CRC, several chemokines, such as CXCL12, CXCL11, and M-CSF secreted by CAFs, have emerged as potential tumor biomarkers. For instance, oxaliplatin has been shown to inhibit CRC progression by suppressing CAF-secreted CXCL11 and the CXCR3/PI3K/AKT pathway [12]. Moreover, CAFs significantly enhance the invasive capacity of CRC cells through the CXCL12/CXCR7 axis [13]. CAFs also secrete M-CSF, IL6, IL8, HGF, and CCL2, which induce the differentiation of a specific macrophage phenotype characterized by high expression of CD163 and CCL2, leading to increased cancer cell invasion in CRC [14]. Nonetheless, the precise mechanisms by which CAFs mediate radioresistance in CRC remain to be fully elucidated.

Emerging evidence indicates that lncRNAs play significant roles in CRC radioresistance [15–17]. For example, our prior study identified a novel transcript, lncRNA SP100-AS1, which was markedly upregulated in CRC tissues resistant to radiation. This lncRNA plays a role in promoting radioresistance and enhancing autophagic flux by acting as a protein “stabilizer”, preventing ATG3 degradation and serving as an RNA “sponge” for miR-622 to maintain ATG3 expression [16]. Additionally, Yu et al. demonstrated that lnc-TLCD2-1 promotes radiation resistance in CRC, potentially by regulating YY1/NF-κB-p65 through targeting miR-193a-5p [18]. Furthermore, knockdown of lncRNA HOTAIR enhances radiosensitivity by modulating the microRNA-93/ATG12 axis in CRC [19]. However, the involvement of CAF-derived lncRNAs in the development of radioresistance in CRC remains largely unclear.

In recent years, numerous lncRNAs have been discovered to influence cancer metabolism, yet the mechanisms involved are still not fully understood. Hypoxia-inducible factor-1α (HIF-1α), a transcription factor that is activated in response to varying oxygen levels, plays a crucial role in determining whether glucose is metabolized through oxidation or glycolysis [20–22]. Under normal physiological conditions, HIF-1α is rapidly hydroxylated by prolyl hydroxylase domain 2 (PHD2) and subsequently targeted for degradation by ubiquitin-mediated proteasomal pathways involving von Hippel–Lindau (VHL) [23]. However, various elements within the tumor microenvironment, including hypoxia [20], reactive oxygen species [24], nitric oxide [25], and specific metabolites [26, 27], can affect PHD2’s hydroxylase activity, inhibiting the degradation of HIF-1α. This results in elevated levels of HIF-1α protein and increased aerobic glycolysis in cancer cells [20].

Our study aimed to elucidate how CAFs contribute to radioresistance in CRC cells and to investigate the roles of CAF-induced lncRNAs linked to this phenotype. Ultimately, our goal is to develop rational strategies that combine traditional cytotoxic therapies with targeted interventions aimed at mitigating the protumorigenic activities of both CAFs and lncRNAs.

## Materials and methods

### CRC tissue specimen

The present study included 30 patients, comprising 15 with radiosensitive tumors and 15 with radioresistant tumors, all of whom underwent surgical procedures at the Third Affiliated Hospital of Soochow University between 2020 and 2021. Tissue samples obtained were preserved in liquid nitrogen at −80 °C. Ethical approval for all experiments involving human tissues was granted by the Ethical Committee of the Third Affiliated Hospital of Soochow University in accordance with the Declaration of Helsinki (No. 2020-science-048). Informed consent was obtained from all participants before their inclusion in the study.

### Isolation and culture of primary human CAFs

CAFs were isolated from fresh colorectal cancer specimens using a Human Tumor Dissociation Kit (Miltenyi Biotec). The tissues were finely minced and subsequently digested. After being filtered through 70 μm cell strainers, the stromal fraction was obtained by centrifugation at 250 g for 5 min and cultured in DMEM supplemented with 15% fetal bovine serum (FBS). Primary human CAFs were purified using magnetic-activated cell sorting with an anti-fibroblast-specific protein (FSP) antibody (catalog number 51676, Cell Signaling Technology). The purity of CAFs was identified confirmed by immunofluorescence (IF) staining and flow cytometry, which showed positive staining for α-SMA and FAP (Fig. S1A, B).

### RNA sequencing analysis

Total RNA was extracted from HCT116 cells treated with conditioned medium from both radioresistant and radiosensitive CAFs, and subjected to HiSeq RNA sequencing. Sequence reads were aligned to the human genome using Tophat. For lncRNA analysis, transcriptomes from each dataset were independently assembled with Cufflinks, followed by pooling and merging of all transcriptomes using Cuffmerge. Differential expression analysis was carried out using the DESeq package, with statistical significance defined as *P* < 0.05. Shanghai OE Biotech Co., Ltd. (Shanghai, China) conducted all sequencing and analytical procedures.

### Cell lines

Human-derived colorectal cancer cell strains, including HCT116, SW480, LS174T, CT26, HT29, and LoVo, were sourced from the American Type Culture Collection (ATCC). Cultivation of these cells was conducted in an RPMI-1640 medium supplemented with 10% FBS and 1% penicillin-streptomycin mixture. The cell strains were validated for the absence of mycoplasma and were preserved under regulated conditions at a temperature of 37 °C in an atmosphere containing 5% CO_2_.

### Xenograft mouse model

In the context of xenograft mouse experimentation, all procedures were in strict accordance with the ethical guidelines established by the Animal Experiments Ethical Committee of the Third Affiliated Hospital of Soochow University. A quantity of 5 × 10^6^ HCT116 cells that had undergone transfection were implanted subcutaneously into 6-week-old male athymic mice; at least five nude mice were used per group. The animals were randomly allocated into two cohorts: one without irradiation and the other with a 2 Gy irradiation dose. When the tumor volumes approximated 90 mm³, the irradiated group received localized irradiation at 2 Gy dosage every 2 days for a period of 10 days. The progression of tumor size was tracked every 5 days, with volumes determined using the formula: (length × width squared)/2. All animal experiments were performed following institutional guidelines for the care and use of laboratory animals. Mice were randomly assigned to different treatment groups to minimize selection bias. In addition, investigators performing tumor measurement and data analysis were blinded to the group allocation throughout the study to ensure objectivity and reduce experimental bias.

### Irradiation

Irradiation treatment was administered at specified doses using a linear accelerator (Varian 2300EX, Varian, Palo Alto, CA) that emitted 6MV X-rays. In summary, monolayer cells received exposure to X-ray radiation over various durations, and were collected immediately after irradiation. Control group cells were subjected to the same conditions in the X-ray generator but did not undergo irradiation.

### Clonogenic cell survival assay

This experiment followed previously established protocols [28]. Cells after transfection were seeded in triplicate into six-well plates and exposed to the specified radiation doses (0, 2, 4, 6, and 8 Gy). Colonies containing more than 50 cells were counted after a 2-week period. Survival curves were fitted using the linear-quadratic model, and the survival fraction was calculated as the number of colonies divided by the number of cells plated × plating efficiency. All data points in the survival curves represented the mean ± SD from at least three independent biological replicates, and each condition was performed in triplicate.

### Transfection

Targeted siRNA sequences were introduced into SW480 and HCT116 cells utilizing Lipofectamine 2000 reagent (Thermo Fisher) as per the manufacturer’s instructions. The cells were subsequently harvested 48 h post-transfection for further experimental procedures. The specific siRNA sequences, custom-designed by GenePharma Biological Technology, can be found in Table S1.

### Apoptosis assay

For apoptosis analysis, CRC cells were cultured in 6-well plates for 24 h prior to transfection with designated siRNA or plasmids. Post-transfection, a period of 24–36 h elapsed before the cells were harvested, rinsed thrice with chilled phosphate-buffered saline (PBS), and carefully resuspended in a 500 μL volume of binding buffer. Subsequently, the cells underwent staining with Annexin V conjugated to FITC, followed by the incorporation of PI for a 10-min incubation period in the dark. The fluorescently labeled cells were subsequently subjected to analysis using a CytoFLEX flow cytometer from BECKMAN COULTER.

### Real-time qPCR (RT-qPCR)

The extraction of total RNA from the cells was facilitated using the Trizol reagent, adhering to the manufacturer’s guidelines. The synthesis of complementary DNA (cDNA) was initiated with the aid of the Superscript First-Strand cDNA Synthesis Kit (catalog number 18080-051, Invitrogen). The qPCR reactions were conducted utilizing the Power SYBR Green PCR Master Mix (Thermo Fisher) on a LightCycle 480 Real-Time PCR System from Roche. A comprehensive list of the primer sequences is presented in Table S1.

### Western blot

The cells were initially washed with ice-cold PBS and then subjected to lysis in RIPA buffer, fortified with a blend of protease and phosphatase inhibitors from CST and PMSF. The lysate was clarified by centrifugation to eliminate insoluble components. Protein concentrations were quantified using the Pierce BCA Protein Assay Kit (Thermo Fisher). Equalized protein samples were resolved through SDS-PAGE and electrophoretically transferred onto polyvinylidene fluoride (PVDF) membranes from Bio-Rad. The membranes were then incubated with a panel of primary antibodies targeting various proteins, including γ-H2AX, HIF-1α, hydroxyl-HIF-1α, PHD2, VHL, DDK-tag, His-tag, ubiquitin, and GAPDH, at the specified dilutions and overnight at 4 °C. The immunoblots were visualized using a Licor Odyssey imaging system, with the relative band intensities standardized to the housekeeping protein GAPDH.

### Bio-plex multiplex immunoassays

The profiles of cytokines released by CAFs were assessed in culture supernatants *via* the Bio-Plex Pro Human Cytokine 17-plex Assay and the Bio-Plex Pro TGF-β1 3-plex Assay (Bio-Rad, Hercules, CA), in accordance with the manufacturer’s guidelines. Cytokines exhibiting significant expression differences were identified.

### Enzyme-linked immunosorbent assay (ELISA)

To measure extracellular TGF-β1 levels, the culture medium was collected and analyzed using a TGF-β1 ELISA kit (Abcam) per the manufacturer’s instructions. Results were reported in ng/mL.

### Immunoprecipitation

Cells underwent lysis in a buffer specifically formulated for immunoprecipitation, which was fortified with a cocktail of protease inhibitors to prevent unwanted degradation. For the IP process, an antibody specific to PHD2 (diluted 1:200; catalog number 19886-1-AP, Proteintech) was mixed with the cell lysate and incubated under refrigerated conditions at 4 °C overnight. Normal rabbit or mouse IgG (ranging from 1–5 μg) was utilized as a non-specific control. Subsequently, Dynabeads Protein A/G (catalog numbers 10002D/10003D, Invitrogen) were added to the mixture and allowed to interact for 1 h at ambient temperature.

### RNA immunoprecipitation (RIP)

The RIP technique was executed in accordance with the instructions provided with the Magna RIP RNA-Binding Protein Immunoprecipitation Kit (17-700, Millipore).

### RNA pull-down assay

The synthesis of biotin-tagged RNA molecules was achieved using the MEGAscript T7 High Yield Transcription kit (Invitrogen), with Bio-16-UTP incorporated to enable biotinylation. To ensure the formation of the correct secondary structure, the biotinylated RNA (1 μg) was prepared in an RNA structure buffer and subjected to a thermal cycling process that included heating at 95 °C for 2 min, followed by cooling on ice and incubation at room temperature. The structured RNA was then combined with a cytoplasmic extract from breast cancer cells or recombinant protein in RIP buffer and incubated for 1 h. Dynabeads M-280 Streptavidin (catalog number 60210, Invitrogen) were added to capture the RNA-protein complexes, followed by further incubation. The beads were washed and boiled, after which they were analyzed using mass spectrometry or western blot.

### In vitro binding assay

For this analysis, DDK-tagged PHD2 (product number TP315158, Origene) and His-tagged HIF-1α (product number ab154478, Abcam) were employed. The potential binding interactions were assessed in the same IP lysis buffer used earlier. Equal amounts of the tagged proteins and folded WARS2-IT1 were combined and incubated at room temperature for 1 h. The resulting protein complexes were then captured through immunoprecipitation, followed by detection and analysis *via* western blot.

### Chromatin immunoprecipitation (ChIP)

For ChIP experiments, we used the Magna ChIP HiSens Chromatin IP Kit (Merck Millipore) according to the specified protocol. Cells treated with TGF-β1 for 2 h were cross-linked with 1% formaldehyde at 37 °C for 10 min, quenched with glycine, and sonicated to produce DNA fragments ranging from 300 to 600 bp using an Ultrasonic Cell Disruptor (Diagenode). Immunoprecipitation was performed using an anti-SMAD3 antibody (catalog number 9523, Cell Signaling Technology). The interaction between the WARS2-IT1 promoter and SMAD3 or IgG was quantified *via* RT-qPCR. Details of the primer sequences used in the ChIP assays can be found in Table S1.

### Luciferase reporter assay

To investigate the regulatory role of SMAD3 on WARS2-IT1 transcription, we cloned the WARS2-IT1 promoter region (−1 to −2000) into the pGL3-enhancer vector. Mutations were introduced within the promoter sequence to identify the Smad3 binding sites (mut1: −844 to −835; mut2: −779 to −770), and these constructs were inserted into the pGL3-enhancer vector. HEK293T cells were transiently transfected with promoter constructs, and after 24 h, luciferase activity was measured and normalized to Renilla luciferase activity using the Dual-Luciferase Reporter Assay System (Promega).

### Statistical analysis

Statistical significance between groups was determined using Student’s *t*-test and one-way ANOVA. Graphs were generated using GraphPad Prism software, with *P* values < 0.05 considered statistically significant. Sample sizes were determined based on previous experience, and all samples were included in the analyses. No sample was excluded from the analyses. Animal subjects were matched for sex, strain, and age, and data analysis was performed in a single-blinded manner. During experimental and outcome assessments, investigators were aware of group assignments.

## Results

### CAF^R^ facilitated radioresistance by activating the expression of WARS2-IT1 in CRC

**Fig. 1:**
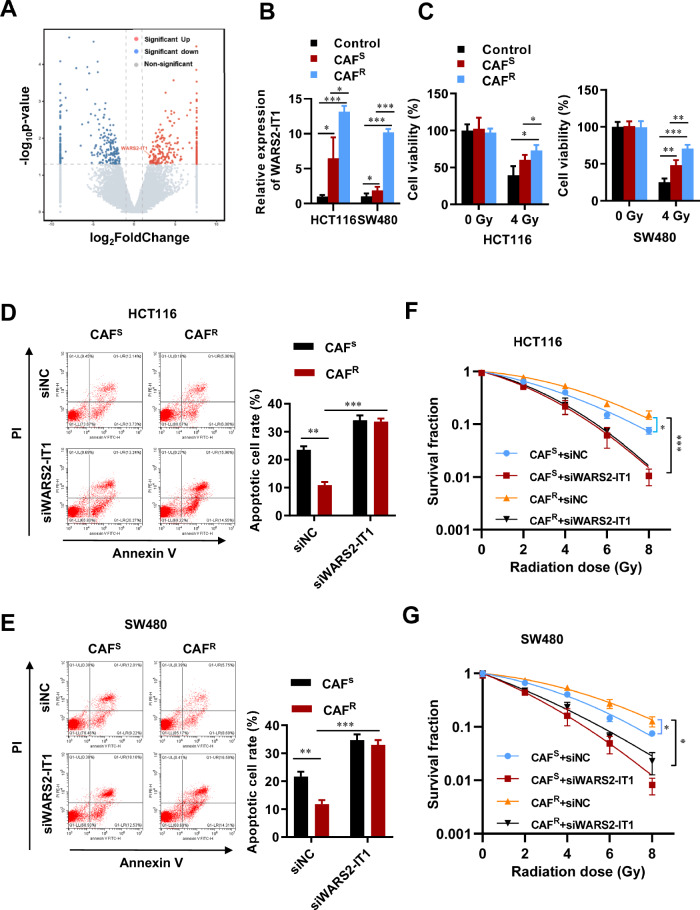
CAF^R^ facilitated radioresistance by activating the expression of WARS2-IT1 in CRC. **A** Whole-transcriptome sequence was performed in HCT116 cells treated with conditioned medium from CAF^S^ (CAF^S^-CM) and CAF^R^ (CAF^R^-CM). Volcano plot showing the differentially expressed lncRNAs between the groups. **B** WARS2-IT1 expression levels in HCT116 and SW480 cells treated with CAF-CM and normal medium. **C** CCK-8 assay were performed to evaluate the radioresistance of CRC cells treated with CAF-CM and normal medium. **D**, **E** Flow cytometry analyses were performed to evaluate the cell apoptosis treated with CAF^S^-CM and CAF^R^-CM without or with WARS2-IT1 siRNA (siWARS2-IT1) or scrambled siRNA (siNC) transfection. **F**, **G** Radiation survival curves were shown for HCT116 and SW480 cells treated with CAF^S^-CM and CAF^R^-CM with or without siRNA transfection. Data are expressed as means ± SD of three independent experiments. N = 3, *P < 0.05, **P < 0.01, ***P < 0.001 compared with the indicated group.

To investigate how CAFs influence the radioresistance of tumor cells, we conducted whole-transcriptome sequencing on HCT116 cells exposed to conditioned medium from radiosensitive CAFs (CAF^S^-CM) and radioresistant CAFs (CAF^R^-CM) isolated from the corresponding CRC patients. Among the 507 differentially expressed lncRNAs identified, 259 were found to be upregulated in CAF^R^-CM treated group (Fig. 1A and Table S2). We then focused on the significantly upregulated lncRNAs for further validation using RT-qPCR. Notably, WARS2-IT1 exhibited a substantial increase in both HCT116 and SW480 cells treated with CAF^S^-CM, and further increased in cells treated with CAF^R^-CM (Fig. 1B). Accordingly, we obtained CRC tissues from patients with varying radiosensitivity characteristics. As illustrated in Fig. S1C, the radioresistant CRC samples demonstrated elevated levels of WARS2-IT1 expression. Furthermore, CRC cell lines exhibited significantly higher endogenous WARS2-IT1 levels when compared to normal epithelial cell lines (Fig. S1D). Interestingly, the treatment with CAF^R^-CM led to a marked enhancement in the viability of HCT116 and SW480 cells at radiation doses below 4 Gy when compared to CAF^S^-CM (Fig. 1C). We also examined the effect of WARS2-IT1 on cell apoptosis. First, we employed two specific siRNAs to knock down WARS2-IT1 in HCT116 and SW480 cells (Fig. S2A), using a scrambled control siRNA (siNC) for comparison. Results from Annexin-V/PI double staining revealed that CRC cells treated with CAF^R^-CM exhibited a significantly reduced apoptotic response compared to those treated with CAF^S^-CM (Fig. 1D, E). Moreover, silencing WARS2-IT1 negated those effects induced by both CAF^R^-CM and CAF^S^-CM (Fig. 1D, E). To further validate the role of CAFs in promoting radioresistance, we performed a clonogenic assay to assess the impact of conditioned medium on the radiosensitivity of HCT116 and SW480 cells. Remarkably, the radioresistance of these cell lines was significantly enhanced following CAF^R^-CM treatment compared to CAF^S^-CM, an effect that was reversed upon downregulation of WARS2-IT1 (Fig. 1F, G and Fig. S3). Collectively, our findings indicated that CAF^R^ promoted radioresistance in CRC cells by upregulating WARS2-IT1 expression.

### WARS2-IT1 knockdown enhances CRC radiosensitivity in vitro

**Fig. 2:**
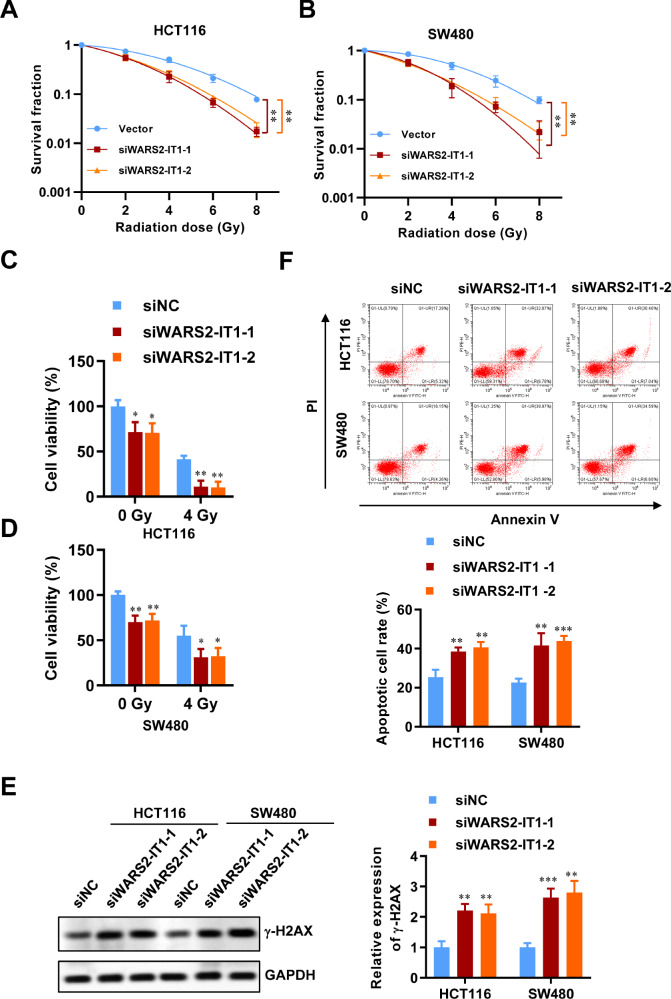
Downregulation of WARS2-IT1 exacerbated radio-induced cell death. **A**, **B** Radiation survival curves of HCT116 and SW480 cells following WARS2-IT1 knockdown using siRNAs. **C**, **D** The cell viability of HCT116 and SW480 cells at a radiation dose of 4 Gy. **E** The expression of γ-H2AX in HCT116 and SW480 cell lines following WARS2-IT1 knockdown and the gray intensity histogram of γ-H2AX expression. **F** Cell apoptosis was evaluated by flow cytometry, and the apoptotic cell percentage was statistically analyzed. Data are expressed as means ± SD of three independent experiments. N = 3, *P < 0.05, **P < 0.01, ***P < 0.001 compared with the indicated group.

To further assess how WARS2-IT1 influences radiosensitivity in CRC cell lines, a clonogenic assay was conducted. Remarkably, downregulation of WARS2-IT1 resulted in a significant enhancement of radiosensitivity in both cell lines (Fig. 2A, B). Furthermore, we examined the viability of HCT116 and SW480 cells following radiation exposure at doses below 4 Gy, revealing that silencing WARS2-IT1 led to decreased cell proliferation and viability (Fig. 2C, D). It is well recognized that radiation therapy induces double-strand breaks in DNA, which must be repaired to allow for continued tumor growth. This underscores the necessity for novel strategies aimed at disrupting DNA repair mechanisms to hinder tumor progression or at least slow it down, ultimately improving patient survival rates. Targeting DNA damage repair is particularly promising for CRC, known for their strong resistance to radiation. Consistent with this, inhibition of WARS2-IT1 resulted in increased expression of γ-H2AX, a key indicator of DNA damage (Fig. 2E). Additionally, we investigated the effect of WARS2-IT1 on apoptosis. Results from Annexin-V/PI double staining indicated that the absence of WARS2-IT1 markedly enhanced the apoptotic response in HCT116 cells compared to controls (Fig. 2F). These findings suggest that WARS2-IT1 plays a critical role in mediating radiation-induced DNA damage and apoptosis in CRC. Therefore, knocking down WARS2-IT1 could enhance the radiosensitivity of CRC cells.

### WARS2-IT1 confers significant radioresistance in vivo

**Fig. 3:**
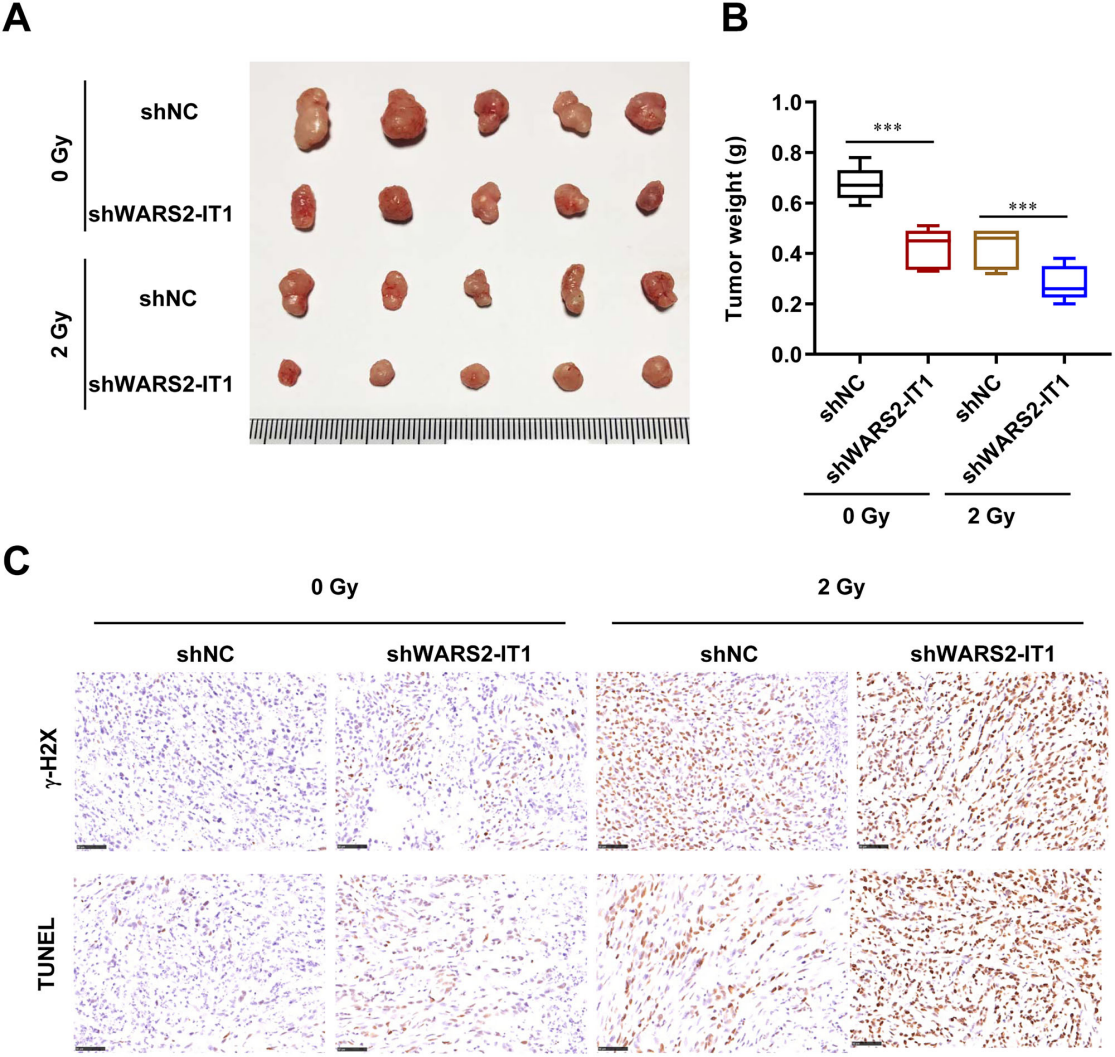
WARS2-IT1 downregulation reduced the growth of irradiated-CRC cells in vivo. **A** WARS2-IT1 was stably knocked down in HCT116, and cells were injected subcutaneously into the axilla of nude mice. 2 Gy IR every other day for 10 days was given until the tumors reached 90 mm^3^. The image of excised tumors is presented. **B** The tumor weight from (**A**). **C** The images following TUNEL staining and representation of relative γ-H2AX expression. Scale bars = 50 μm. Data are expressed as means ± SD. N = 5, *P < 0.05, **P < 0.01, ***P < 0.001 compared with the indicated group.

We next explored the in vivo impact of WARS2-IT1 by creating a xenograft model using HCT116 cells with stable knockdown of WARS2-IT1 (shWARS2-IT1) or an empty scrambled siRNA control (shNC), achieved through subcutaneous injections in nude mice. Once the tumors reached a volume of 90 mm³, all mice underwent radiation treatment. As depicted in Fig. 3A, successful stable knockdown of WARS2-IT1 was confirmed *via* lentiviral infection of HCT116 cells, resulting in reduced tumor growth in the xenografts compared to the control group. Furthermore, following irradiation, HCT116 tumors with WARS2-IT1 suppression exhibited slower growth rates than those in the control cohort (Fig. 3A). Post-fractionated radiation treatment, the reduction in tumor weight was more pronounced in CRC tumors where WARS2-IT1 had been silenced (Fig. 3B). At the conclusion of the study, tumor weights were recorded and analyzed. Additionally, a transferase-mediated dUTP nick-end labeling (TUNEL) assay revealed that downregulation of WARS2-IT1 resulted in heightened DNA damage in response to 2 Gy radiation, alongside increased γ-H2AX staining (Fig. 3C). To further clarify the function of WARS2-IT1, we employed two additional CRC cell lines, LoVo and HT29, and obtained similar results (Fig. S4). This indicated that silencing WARS2-IT1 significantly inhibited the growth rate of CRC tumors, particularly under radiation exposure. Overall, these findings suggested that WARS2-IT1 silencing enhanced the radiosensitivity of CRC both in vitro and in vivo.

### WARS2-IT1 stabilizes HIF-1α by blocking PHD2 and HIF-1α interactions

**Fig. 4:**
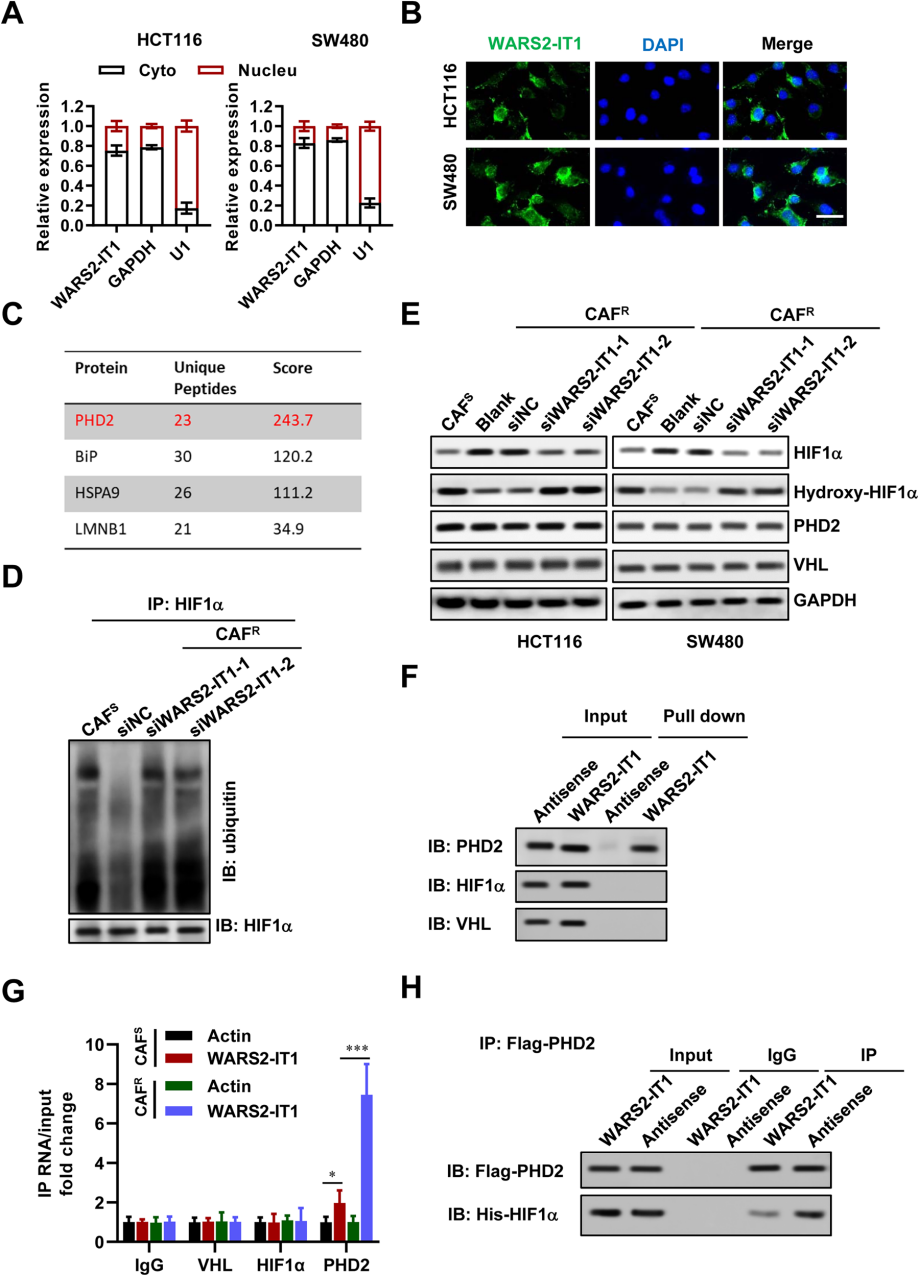
WARS2-IT1 stabilized HIF-1α by blocking PHD2 and HIF-1α interactions. **A** RT-qPCR analysis following subcellular fractionation of WARS2-IT1 of HCT116 and SW480 cells. **B** Representative FISH images showing the cellular localization of WARS2-IT1. The WARS2-IT1 probe was labeled with FAM (green), and the nuclei were stained with DAPI (blue). Scale bar = 50 μm. **C** The top four WARS2-IT1-interacting proteins according to the number of identified unique peptides. **D** HIF-1α ubiquitination in HCT116 cells treated with CAF-CM was assayed by immunoprecipitation and immunoblotting. MG132 (20 μM) was added before protein extraction to inhibit HIF-1α degradation. **E** Western blot of HIF-1α, hydroxy-HIF-1α, PHD2 and VHL. MG132 was added before protein extraction to demonstrate hydroxyl-HIF-1α. RNA pull-down (**F**) and RNA immunoprecipitation (**G**) assays demonstrating the interaction of WARS2-IT1 and PHD2, HIF-1α, or VHL in HCT116 cells treated with CAF^S^-CM and CAF^R^-CM. **H** Immunoprecipitation analysis for the in vitro interaction of WARS2-IT1 and PHD2 or HIF-1α. Data are expressed as means ± SD of three independent experiments. N = 3, *P < 0.05, **P < 0.01, ***P < 0.001 compared with the indicated group.

We performed cellular fractionation PCR to determine the localization of WARS2-IT1 in CRC cells, revealing that it predominantly resides in the cytoplasm (Fig. 4A). This finding was corroborated by RNA fluorescence in situ hybridization (RNA-FISH), suggesting that WARS2-IT1 plays a role in regulating downstream pathways at the post-transcriptional level (Fig. 4B). Given their limited protein-coding capability, many lncRNAs often exert their effects through interactions with proteins [29–31]. To investigate how CAFs induce WARS2-IT1 and contribute to radioresistance in CRC, we conducted an RNA pull-down assay followed by mass spectrometry, identifying PHD2 as a binding partner for WARS2-IT1 (Fig. 4C).

Consistent with the observed extended half-life of HIF-1α (data not shown), we found that the hydroxylation and subsequent degradation of HIF-1α were significantly augmented in WARS2-IT1-knockdown HCT116 cells treated with CAF^R^-CM (Fig. 4D, E), indicating an inhibition of the functions of PHD2 and VHL in these conditions. However, PHD2 expression levels remained consistent across CRC cells treated with either CAF^S^-CM or CAF^R^-CM, regardless of WARS2-IT1 status, suggesting minimal effect on PHD2 expression or stability (Fig. 4E). Under normal physiological conditions, PHD2 rapidly hydroxylates HIF-1α, leading to its ubiquitin-mediated proteasomal degradation through VHL. Our analysis showed that VHL protein levels did not significantly change under various treatments. Nonetheless, WARS2-IT1 knockdown contributed to enhanced hydroxylation and degradation of HIF-1α in CRC cells (Fig. 4E). Further, the RNA pull-down assay confirmed that WARS2-IT1 interacted specifically with PHD2, rather than HIF-1α or VHL (Fig. 4F). Additionally, RNA immunoprecipitation using anti-PHD2 successfully retrieved WARS2-IT1, while anti-HIF-1α and anti-VHL did not (Fig. 4G). Co-localization studies *via* FISH and immunostaining demonstrated that WARS2-IT1 and PHD2 were present together in the cytoplasm of CRC cells (Fig. S5).

Next, we examined how WARS2-IT1 disrupted the interaction between PHD2 and HIF-1α using an ex vivo system with recombinant proteins and in vitro transcribed lncRNAs [32]. Notably, the introduction of WARS2-IT1, but not an antisense lncRNA, effectively disrupted the PHD2-HIF-1α interaction (Fig. 4H). Moreover, deletion mapping analysis identified a 188–375 nt region of WARS2-IT1 that was required for the interaction between WARS2-IT1 and PHD2 (Fig. S6). Collectively, our findings indicated that WARS2-IT1 bound to PHD2, inhibiting its association with HIF-1α, thereby preventing the hydroxylation and degradation of HIF-1α. Given HIF-1α‘s critical role in glycolysis regulation, we proceeded to analyze the effects of CAF^S^-CM and CAF^R^-CM, as well as the influence of WARS2-IT1, on glycolytic activity. Using a Seahorse XF24e Extracellular Flux Analyzer, we assessed the extracellular acidification rate (ECAR) in CRC cells treated with CAF^S^-CM and CAF^R^-CM. Our results indicated that CAF^R^-CM significantly enhanced glycolytic activity compared to CAF^S^-CM (Fig. S7A, B). Also, CAF^R^-CM resulted in increased lactate production compared to CAF^S^-CM (Fig. S7C, D). Moreover, silencing WARS2-IT1 resulted in reduced ECAR (Fig. S7E, F) and lactate production (Fig. S7G, H). We then transfected HCT116 and SW480 cells with WARS2-IT1 overexpression plasmids for 48 h and used siRNA to knock down HIF-1α expression (Fig. S2B). Conversely, overexpression of WARS2-IT1 led to increased ECAR, while HIF-1α knockdown in these cells negated the pro-glycolytic effects of WARS2-IT1 (Fig. S8). These findings underscored the role of WARS2-IT1 in promoting tumor growth by enhancing HIF-1α-related glycolytic signaling.

### WARS2-IT1 increases CRC cell radioresistance activity through HIF-1α

**Fig. 5:**
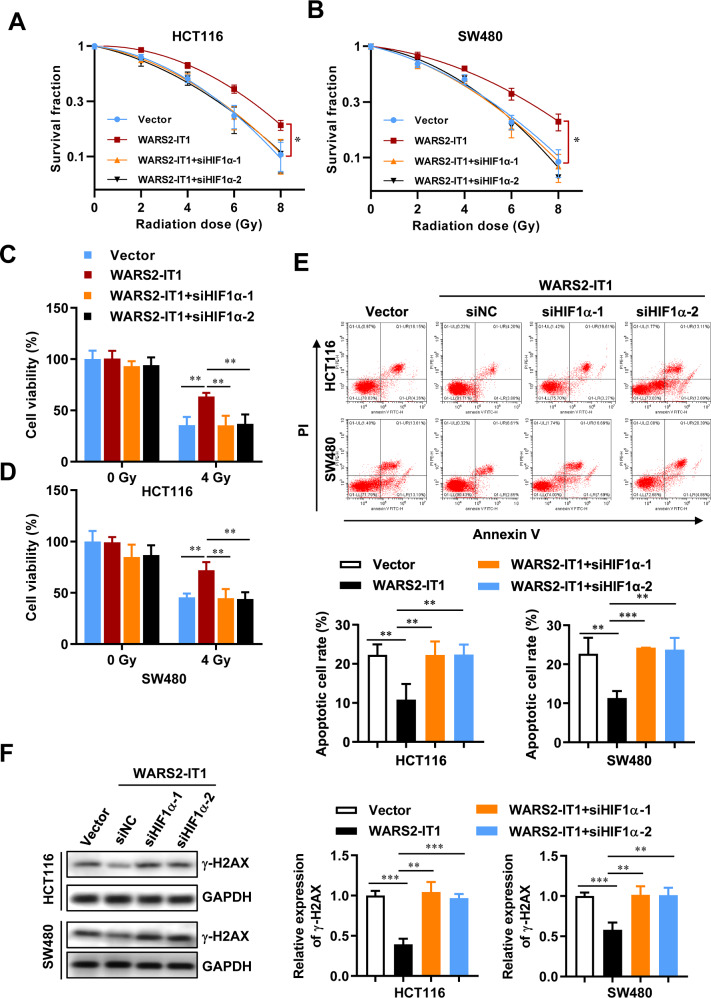
CAF-induced WARS2-IT1 upregulation significantly increased the radioresistance *via* HIF-1α. Radiation survival curves are shown for HCT116 cells (**A**) and SW480 cells (**B**) that were transfected with vectors containing WARS2-IT1 before the transfection with siRNA for HIF-1α. **C**, **D** The cell viability of HCT116 and SW480 cells at a radiation dose of 0 Gy and 4 Gy. **E** Cell apoptosis was evaluated by flow cytometry, and the apoptotic cell percentage was statistically analyzed. **F** The expression of γ-H2AX in HCT116 and SW480 cell lines that were transfected with vectors containing WARS2-IT1 before the transfection with siRNA for HIF-1α. Data are expressed as means ± SD of three independent experiments. *N* = 3, *P < 0.05, **P < 0.01, ***P < 0.001 compared with the indicated group.

In line with our findings, we detected elevated levels of HIF-1α protein in CRC cells treated with CAF^R^-CM compared to those receiving CAF^S^-CM (Fig. 4E). This increase was linked to an extended half-life of the HIF-1α protein, while its mRNA expression remained stable (data not shown). We further determined whether silencing HIF-1α could reverse the radioresistance conferred by WARS2-IT1. As illustrated in Fig. 5A, B, radiation clonogenic survival assays revealed that WARS2-IT1 overexpression of CRC cells exhibited significant radioresistance when compared to cells transfected with control vectors. However, the knockdown of HIF-1α effectively reversed this WARS2-IT1-mediated radioresistance.

Furthermore, WARS2-IT1 overexpression led to an increase in cell viability following ionizing radiation treatment and reduced apoptosis induced by irradiation; these effects were negated when HIF-1α was knocked down (Fig. 5C–E). Additionally, WARS2-IT1 mitigated the rise in γ-H2AX expression post-irradiation, whereas HIF-1α knockdown resulted in an increase in γ-H2AX levels (Fig. 5F). Collectively, these results suggested that WARS2-IT1 enhanced radioresistance in CRC cells by stabilizing the HIF-1α protein.

### CAFs induce the expression of the WARS2-IT1 in CRC cells via paracrine TGF-β1

**Fig. 6:**
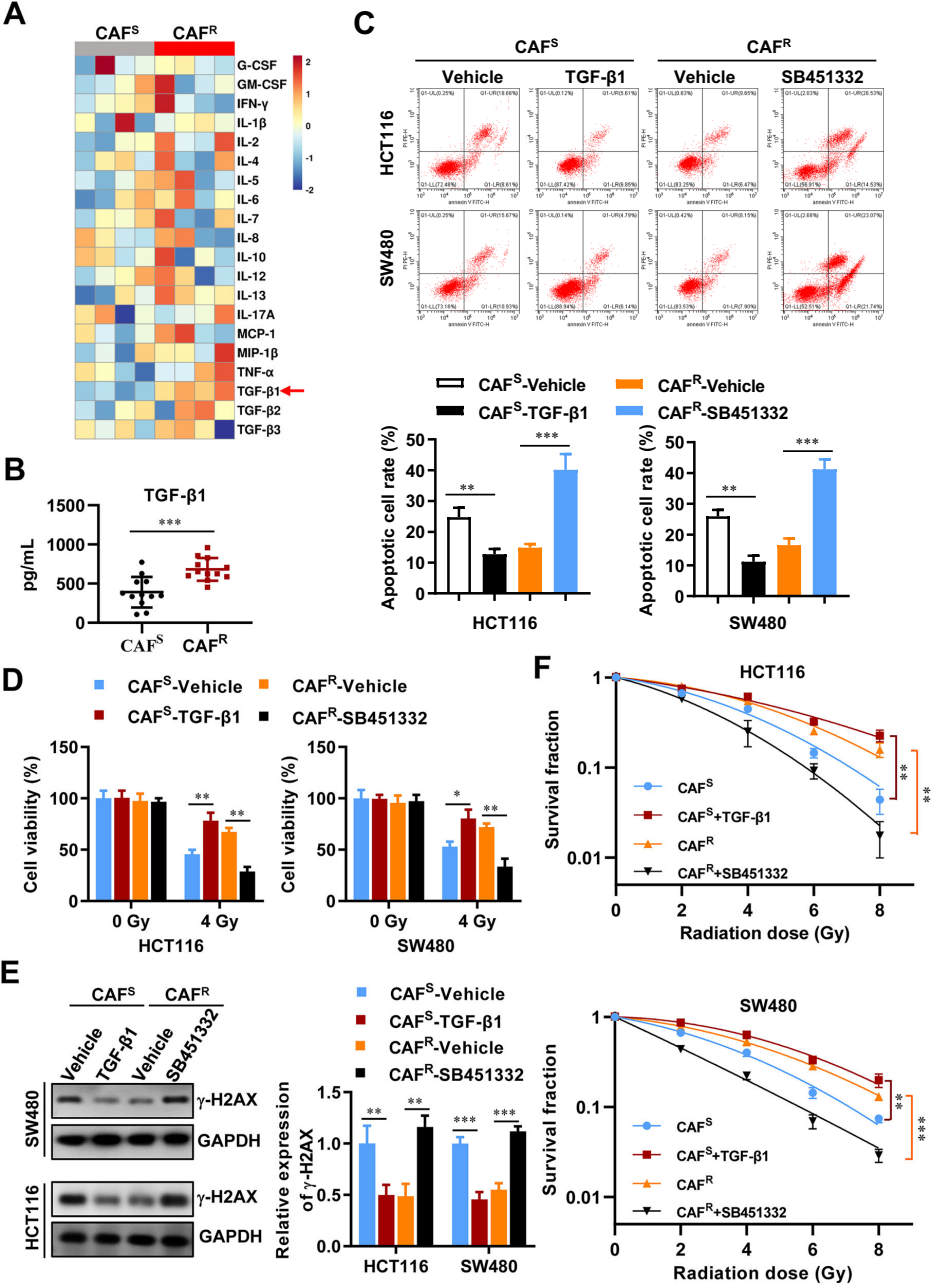
Paracrine TGF-β1 was essential for CAF^R^-induced radioresistance. **A** Heatmap of 20 different human cytokine expression levels in CAF^S^-CM and CAF^R^-CM using Bio-Plex Pro Human Cytokine 17-plex and Bio-Plex Pro TGF-β1 3-plex immunoassay. **B** ELISA analysis of the TGF-β1 levels in CAF^S^-CM and CAF^R^-CM from 24 radioresistant and radiosensitive patients. Cell apoptosis (**C**), cell viability (**D**), expression of γ-H2AX (**E**), and radiation survival curves (**F**) were examined to evaluate the radioresistance of CRC cells treated with CAF^S^-CM (in the absence or presence of TGF-β1) and CAF^R^-CM (with or without TGF-β1 specific inhibitor SB451332). Data are expressed as means ± SD of three independent experiments. N = 3, *P < 0.05, **P < 0.01, ***P < 0.001 compared with the indicated group.

To elucidate the mechanisms by which CAFs contribute to radioresistance, we analyzed the cytokine profiles of CAF^R^-CM and CAF^S^-CM using the Bio-Plex Pro Human Cytokine 23-Plex immunoassay. This analysis revealed a set of cytokines that were significantly elevated in CAF^R^-CM. Among these, the serum concentration of transforming growth factor-β (TGF-β1), known for its role in inducing chemoresistance [33], was markedly higher in CAF^R^-CM compared to CAF^S^-CM (Fig. 6A). We then conducted an ELISA to quantify TGF-β1 levels in CAF^R^-CM and CAF^S^-CM samples obtained from 24 patients with varying degrees of radioresistance. As anticipated, CAF^R^ produced substantially more TGF-β1 than CAF^S^ (Fig. 6B).

Subsequently, we investigated the impact of TGF-β1 on the radioresistance of CRC cells induced by CAFs. Our findings showed that treatment with TGF-β1 resulted in comparable enhancements in radioresistance in CRC cells exposed to CAF^S^-CM, similar to those treated with CAF^R^-CM (Fig. 6C–F). Moreover, the use of small-molecule inhibitors targeting TGF-β1, such as SB451332, effectively negated the radioresistance-promoting effects of CAF^R^-CM (Fig. 6C–F). Taken together, these results indicated that TGF-β1 was primarily secreted by CAF^R^, and inhibiting its function could reverse the radioresistance conferred by CAF^R^ in cancer cells.

### CAFs transactivate WARS2-IT1 by TGF-β1 via SMAD3-dependent mechanism

**Fig. 7:**
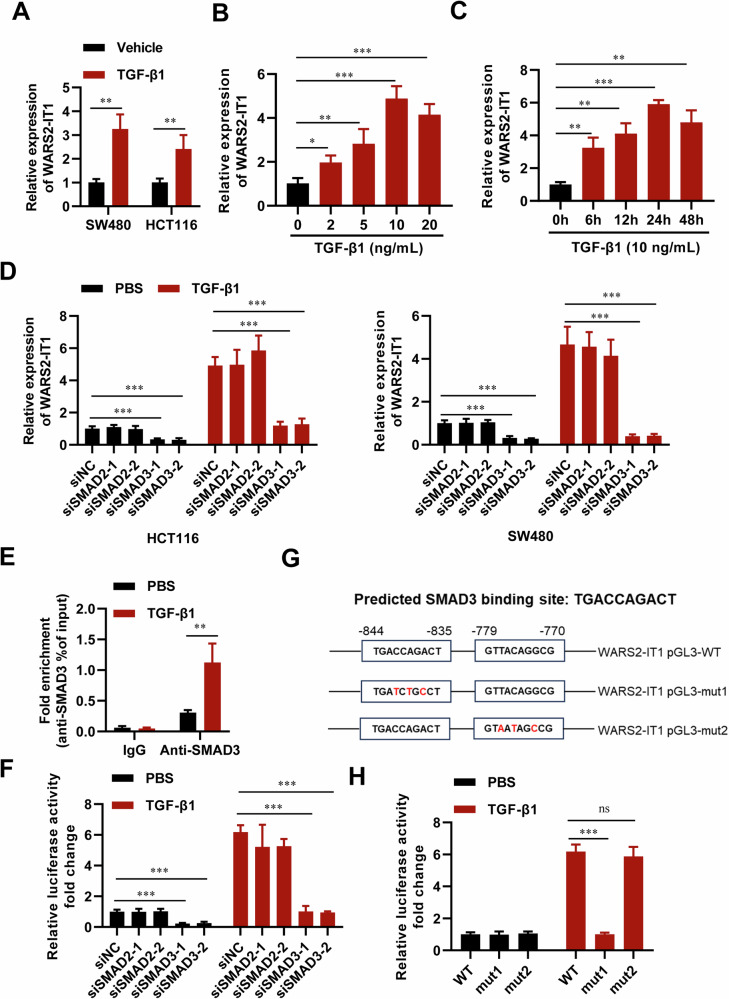
WARS2-IT1 was regulated by TGF-β1 *via* SMAD3-dependent mechanism. **A** Expression of WARS2-IT1 assayed by RT-qPCR. **B**, **C** Expression of WARS2-IT1, assayed by RT-qPCR, was upregulated by TGF-β1 in a dose- and time dependent manner in HCT116 cells. **D** Knocking down SMAD3 decreased the expression of WARS2-IT1 assayed by RT-qPCR in CRC cells with or without TGF-β1 stimulation. **E** ChIP assay showing that SMAD3 bound to the WARS2-IT1 promoter under TGF-β1 stimulation. **F** Knocking down SMAD3 decreased the transcription of WARS2-IT1, as detected by luciferase reporter assays. **G** Mutation strategies for the WARS2-IT1 promoter. The underlined nucleotides indicate the mutated nucleotides. **H** Mutation of SMAD3-binding site 1 reduced WARS2-IT1 transcription in luciferase reporter assays. As a control, an equivalent amount of PBS was added to the culture medium. Data are expressed as means ± SD of three independent experiments. ns indicates nonsignificant. N = 3, *P < 0.05, **P < 0.01, ***P < 0.001 compared with the indicated group.

RT-qPCR analyses confirmed that WARS2-IT1 was consistently expressed in HCT116 and SW480 cells and showed an increase following TGF-β1 stimulation (Fig. 7A). The upregulation of WARS2-IT1 in response to TGF-β1 was found to be both dose- and time-dependent (Fig. 7B, C). Given the increase in WARS2-IT1 expression caused by TGF-β1, we explored the involvement of SMAD2 and SMAD3, crucial components of the TGF-β1 signaling pathway, in regulating its transcription. We observed a decline in WARS2-IT1 levels upon silencing SMAD3 with siRNAs, while knockdown of SMAD2 had no effect on WARS2-IT1 expression (Fig. 7D). This indicated that the transcription of WARS2-IT1 was dependent on SMAD3.

To further investigate this interaction, we conducted chromatin immunoprecipitation (ChIP) analysis using an anti-SMAD3 antibody to assess whether SMAD3 bound to the promoter of WARS2-IT1. Our results from ChIP analysis confirmed that SMAD3 indeed interacted with the promoter region of WARS2-IT1, establishing it as a transcriptional target of SMAD3 (Fig. 7E). According to JASPAR, a transcription factor binding profile database [34], two potential SMAD3-binding sites were predicted within the WARS2-IT1 promoter region (−844 to −835 bp and −779 to −770 bp in the human genome build hg38). To validate these predictions, we cloned the promoter segment from −1 to −1000 bp into a pGL3-enhancer vector. The luciferase reporter assay revealed that silencing SMAD3 significantly decreased WARS2-IT1 promoter activity, whereas silencing SMAD2 did not have this effect (Fig. 7F). Furthermore, we created several pGL3 reporter constructs containing either mutant or wild-type sequences for the regions between −844 to −835 bp and −779 to −770 bp (Fig. 7G). Mutating the SMAD3-binding site (−844 to −835 bp) led to reduced luciferase activity; however, altering the downstream sequence (−779 to −770 bp) did not affect transcriptional activity (Fig. 7H). These findings suggested that SMAD3 directly bound to the WARS2-IT1 promoter and enhanced its expression.

## Discussion

Radiotherapy is a widely adopted non-surgical treatment for patients with advanced colorectal cancer (CRC) [35]. Within this therapeutic context, the tumor microenvironment-particularly cancer-associated fibroblasts (CAFs)-has emerged as a key determinant of radioresistance through a variety of mechanisms [6]. Our research aimed to clarify the role of factors originating from CAFs, particularly focusing on a specific subset known as CAF^R^, which was derived from patients exhibiting radioresistant CRC. CAFs represent a heterogeneous group existing within the tumor stroma, displaying a range of phenotypic and functional attributes that affect tumor dynamics and responses to therapy [36]. Notably, our results revealed that CAF^R^ secreted markedly higher levels of TGF-β1 than CAF^S^ derived from radiosensitive CRC patients, suggesting that this variation in TGF-β1 secretion may be a critical driver of enhanced radioresistance.

An expanding body of evidence underscores the profound impact of the tumor microenvironment on CRC progression. Resident stromal cells, including fibroblasts and immune cells, engage in dynamic interactions with cancer cells to regulate disease advancement. Among the pivotal mediators of these interactions is the immunoregulatory cytokine TGF-β1—a multifunctional molecule known for its dual roles in cancer, acting as both a tumor suppressor and a promoter depending on the tumor stage and microenvironmental context [37, 38]. In CRC, TGF-β1 signaling is associated not only with tumor progression but also with alterations in radiotherapy response, potentially leading to increased radioresistance. Elucidating how CAFs and TGF-β1 contribute to CRC radioresistance is essential for devising effective therapeutic approaches. Recent investigations have started to uncover these complexities, identifying distinct subsets of CAFs that play a role in therapy resistance [39]. Notably, CAFs isolated from tumors of radioresistant CRC patients show phenotypic and functional distinctions when compared to those from radiosensitive tumors, suggesting their active participation in modulating the adaptive responses of tumors to radiotherapy.

In addition, growing evidence indicates that long noncoding RNAs (lncRNAs) are critical mediators of the cross-talk between immune cells and cancer cells, thereby influencing tumor progression. LncRNAs, which regulate gene expression through mechanisms such as chromatin remodeling, transcriptional modulation, and post-transcriptional processing, have been implicated in CRC radioresistance [40]. Specific lncRNAs modulate key cellular processes, including DNA damage repair, apoptosis, and cell cycle control, which determine cellular responses to radiation [16, 19, 41, 42]. A central focus of our investigation was to examine how lncRNAs mediate CAF-induced radioresistance. Through comprehensive profiling of CRC cells exposed to CAF-conditioned medium, we identified WARS2-IT1 as a crucial regulator activated by TGF-β1 signaling. Mechanistically, WARS2-IT1 enhances radioresistance by disrupting the interaction between PHD2 and HIF-1α, thereby inhibiting the hydroxylation and subsequent proteasomal degradation of HIF-1α under normoxic conditions. The resulting stabilization of HIF-1α promotes an increase in glycolytic flux—a metabolic shift that is closely linked to radioresistance. These findings provide novel insights into the molecular mechanisms by which CAF-induced WARS2-IT1 contributes to the adaptive responses of CRC cells to radiotherapy.

**Fig. 8:**
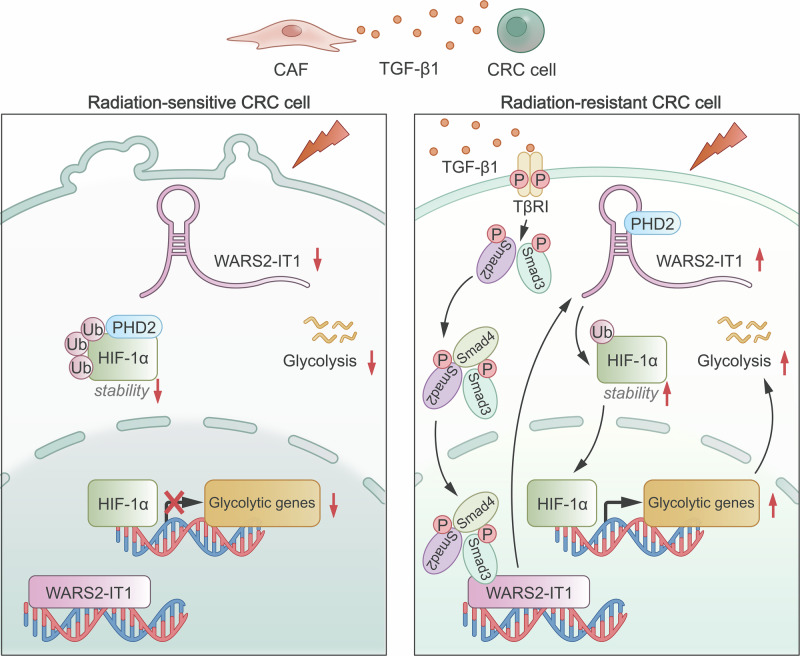
Schematic illustration depicting a proposed model of the molecular mechanism of WARS2-IT1 in initiating radioresistance in human CRC.

In summary, our identification of WARS2-IT1 as a pivotal mediator of radioresistance underscores its potential as a therapeutic target in CRC. Strategies aimed at inhibiting or modulating WARS2-IT1 expression could improve the radiosensitivity of CRC cells by countering the resistance mechanisms orchestrated by CAF-derived factors, such as TGF-β1. Targeting these specific components of the tumor microenvironment presents a promising approach to enhance the efficacy of radiotherapy for CRC. Overall, our study sheds light on the critical role of CAFs and their secreted factors in promoting radioresistance, while establishing WARS2-IT1 as a key element in TGF-β1-mediated pathways. These insights pave the way for the development of innovative therapeutic strategies designed to disrupt CAF-induced adaptive responses and improve treatment outcomes for CRC patients resistant to standard radiotherapy (Fig. 8).

## Supplementary information

Supplementary legends

Figure S1

Figure S2

Figure S3

Figure S4

Figure S5

Figure S6

Figure S7

Figure S8

Table S1

Table S2

Full and uncropped western blots

## Data Availability

All data needed to evaluate the conclusions in the paper are available upon request.
