# Supplementary material for: Cancer-associated fibroblast-induced lncRNA WARS2-IT1 confers radioresistance of colorectal cancer via enhancing HIF-1α stability
Source: Cell Death Dis. 2025 Nov 10;16(1):823. doi: 10.1038/s41419-025-08058-1 (PMC12603266; doi:10.1038/s41419-025-08058-1)
Supplement: Supplementary file 1 — Supplementary legends [file 41419_2025_8058_MOESM1_ESM.docx]

**Supplementary legends**

**Fig. S1** (A) Immunofluorescence staining of the fibroblast markers α-SMA and FAP for CAFs. (B) Flow cytometry confirmed the expression of CAF markers α-SMA and FAP. (C) The relative expression of WARS2-IT1 in radiotherapy-sensitive (Sensitive, n = 15) and resistant patients’ colorectal tissues (Resistant, n = 15). (D) The relative expression of WARS2-IT1 in normal human colon mucosal epithelial cell line CCD841 and a series of human CRC cell lines. **P* < 0.05, ***P* < 0.01, ****P* < 0.001 compared with the indicated group.

**Fig. S2** RT-qPCR analysis verified the knockdown (A) and overexpression (B) efficiency of WARS2-IT1 in HCT116 and SW480 cells. N = 3, ***P* < 0.01, ****P* < 0.001 compared with the indicated group.

**Fig. S3** Radiation survival curves are shown for HCT116 (A) and SW480 (B) cells treated with CAF^S^-CM and CAF^R^-CM. Data are expressed as means ± SD of three independent experiments. N = 3, **P* < 0.05, compared with the indicated group.

**Fig. S4** Downregulation of WARS2-IT1 exacerbated radio-induced cell death. (A-B) Radiation survival curves of LoVo and HT29 cells following WARS2-IT1 knockdown using siRNAs. (C-D) The cell viability of LoVo and HT29 cells at a radiation dose of 4 Gy. (E) The expression of γ-H2AX in LoVo and HT29 cell lines following WARS2-IT1 knockdown and the gray intensity histogram of γ-H2AX expression. (F) Cell apoptosis was evaluated by flow cytometry, and the apoptotic cell percentage was statistically analyzed. Data are expressed as means ± SD of three independent experiments. N = 3, **P* < 0.05, ***P* < 0.01 compared with the indicated group.

**Fig. S5** Representative images of the colocalization of WARS2-IT1 and PHD2 in HCT116 and SW480 cells. WARS2-IT1 and PHD2 was examined by FISH and immunofluorescence staining, respectively. Scale bars = 50 μm.

**Fig. S6** WARS2-IT1 was physically associated with PHD2. Biotin-labeled RNA transcripts corresponding to various fragments of WARS2-IT1 or its antisense sequence (dotted line) were incubated with whole-cell lysates from HCT116 cells. The RNA-protein complexes were captured using streptavidin-conjugated beads, followed by extensive washing. The presence of PHD2 in the pull-down complexes was subsequently confirmed by western blot analysis.

**Fig. S7** (A-B) The ECAR was measured in HCT116 and SW480 cells treated with CAF^S^-CM and CAF^R^-CM using an XF Extracellular Flux Analyzer. (C-D) The lactate production was measured in HCT116 and SW480 cells treated with CAF^S^-CM and CAF^R^-CM. (E-F) The ECAR was measured in HCT116 and SW480 cells following WARS2-IT1 knockdown using siRNA by an XF Extracellular Flux Analyzer. (G-H) The lactate production was measured in HCT116 and SW480 cells following WARS2-IT1 knockdown. N = 3, **P* < 0.05, ***P* < 0.01, ****P* < 0.001 compared with the indicated group.

**Fig. S8** The ECAR was measured and analyzed in HCT116 cells (A) and SW480 cells (B) that were transfected with vectors containing WARS2-IT1 before the transfection with siRNA for HIF-1α using an XF Extracellular Flux Analyzer. N = 3, **P* < 0.05, ***P* < 0.01, ****P* < 0.001 compared with the indicated group.

**Table S1** Sequences for siRNAs, FISH probe, primer sequences for RT-qPCR and ChIP assay.

**Table S2** RNA-seq analysis identified a total of 507 differentially expressed lncRNAs in radioresistant versus radiosensitive patients, among which 259 lncRNAs were upregulated, and 248 lncRNAs were downregulated.
