# Supplementary figures and images for: Cancer-associated fibroblast-induced lncRNA WARS2-IT1 confers radioresistance of colorectal cancer via enhancing HIF-1α stability
Source: Cell Death Dis. 2025 Nov 10;16(1):823. doi: 10.1038/s41419-025-08058-1 (PMC12603266; doi:10.1038/s41419-025-08058-1)

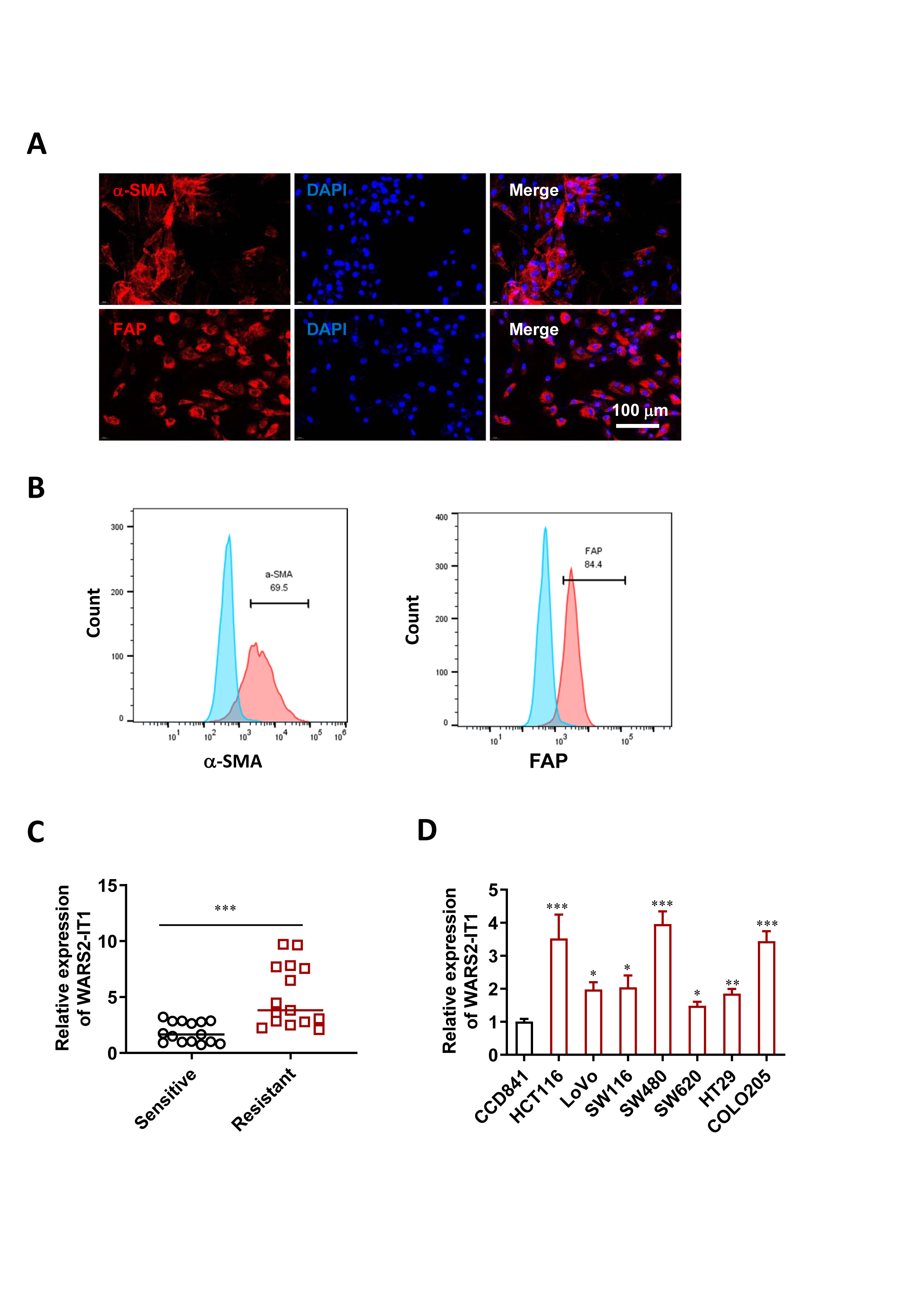

Supplement: Supplementary file 2 — Figure S1 [file 41419_2025_8058_MOESM2_ESM.tif]

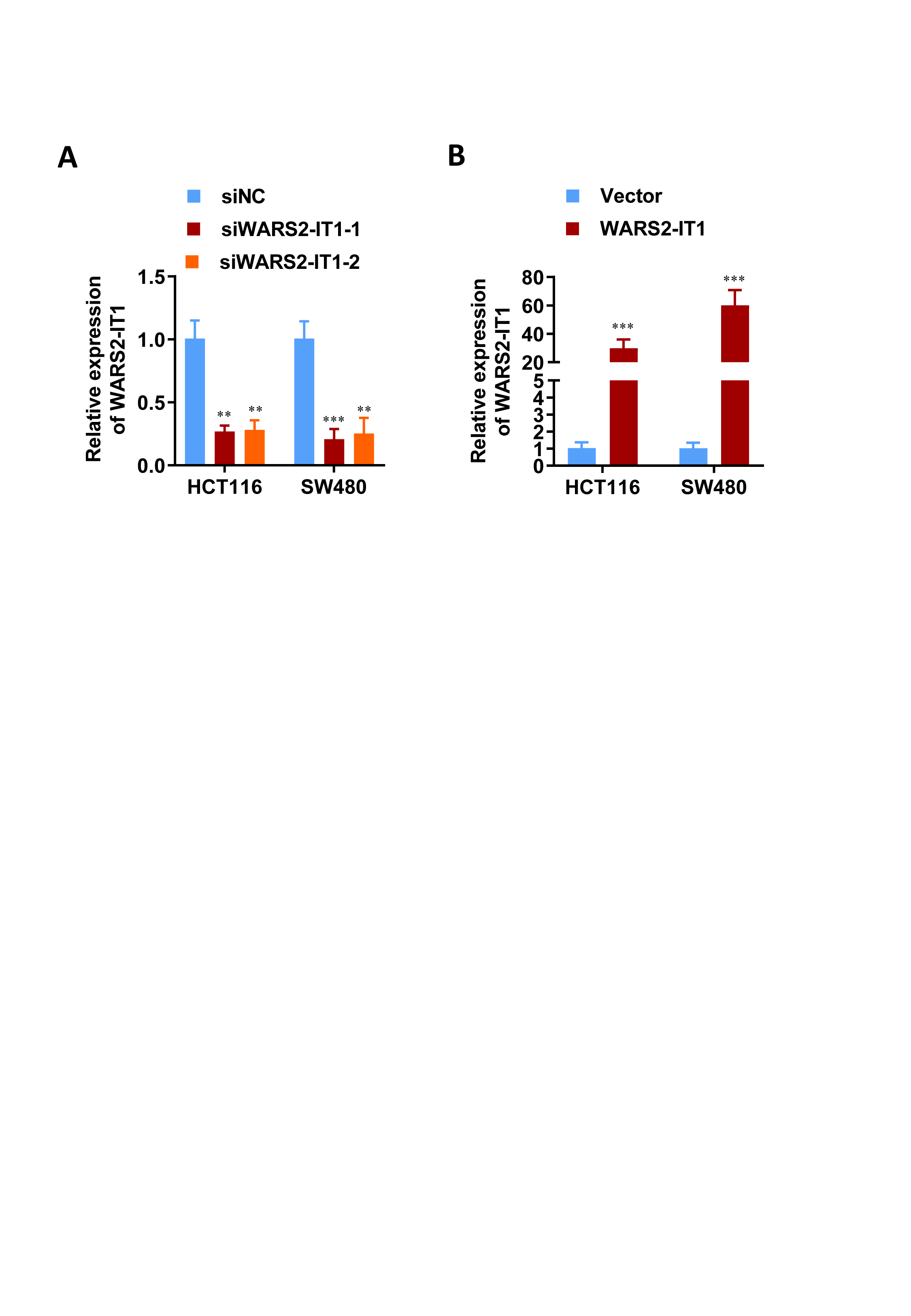

Supplement: Supplementary file 3 — Figure S2 [file 41419_2025_8058_MOESM3_ESM.tif]

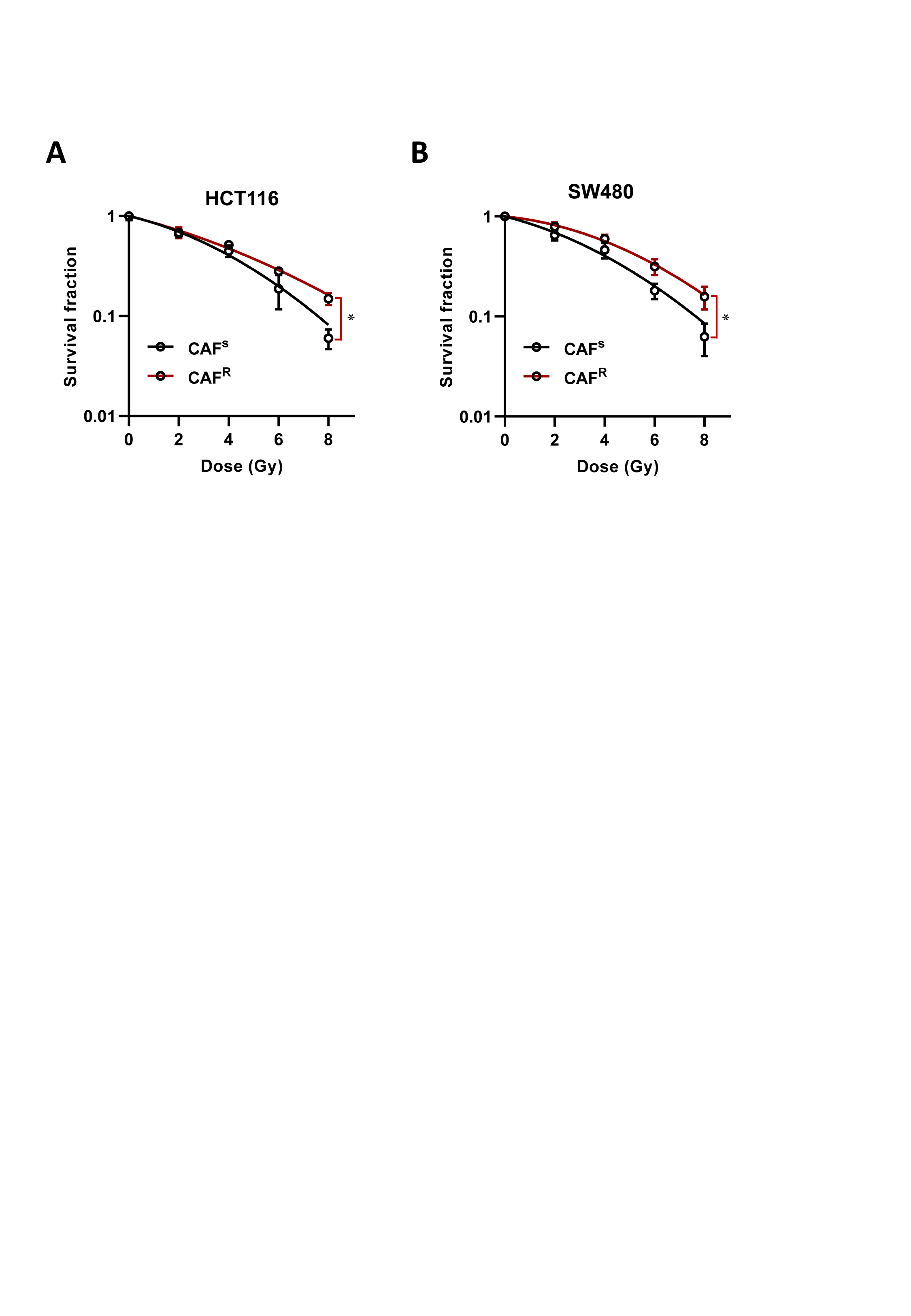

Supplement: Supplementary file 4 — Figure S3 [file 41419_2025_8058_MOESM4_ESM.tif]

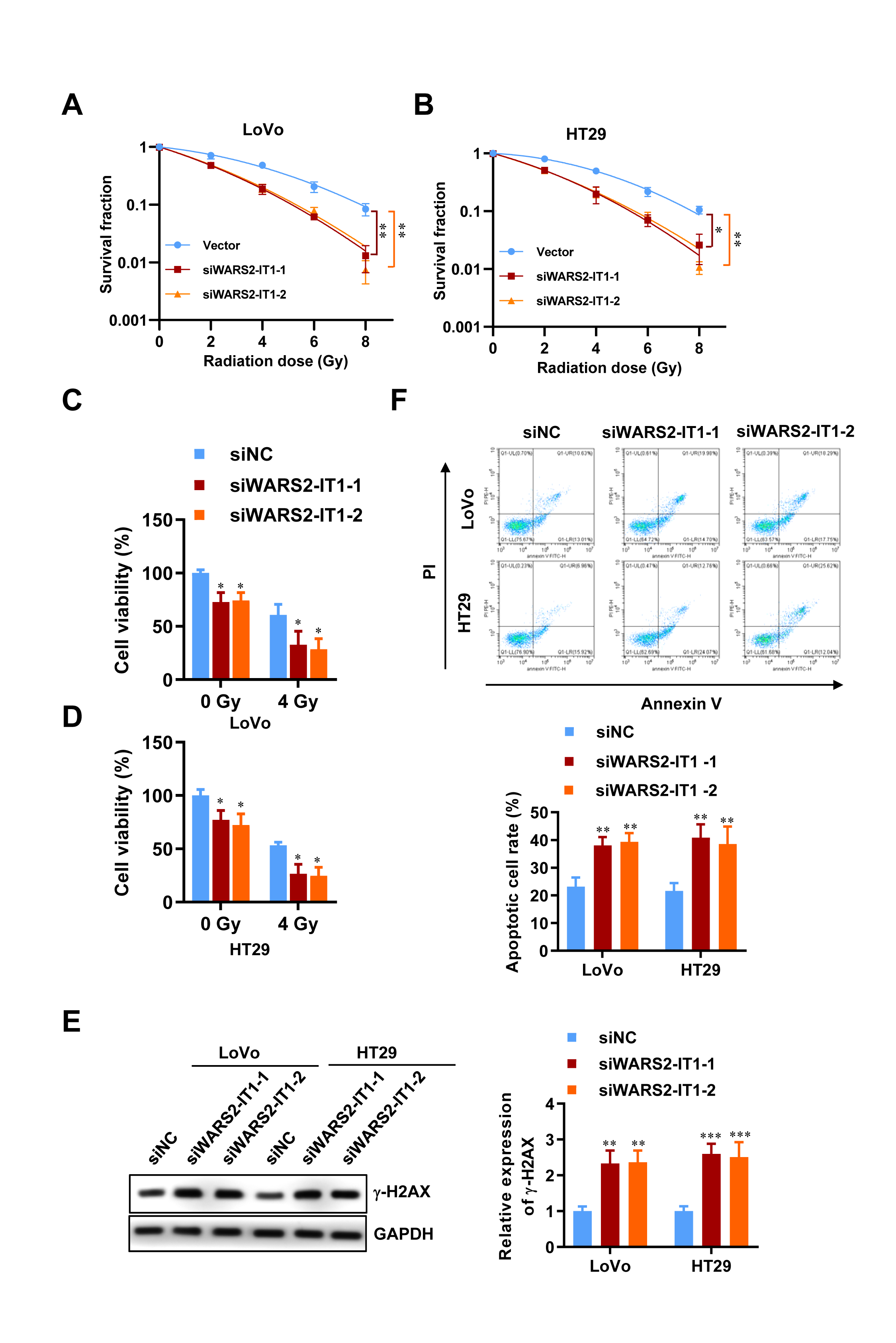

Supplement: Supplementary file 5 — Figure S4 [file 41419_2025_8058_MOESM5_ESM.tif]

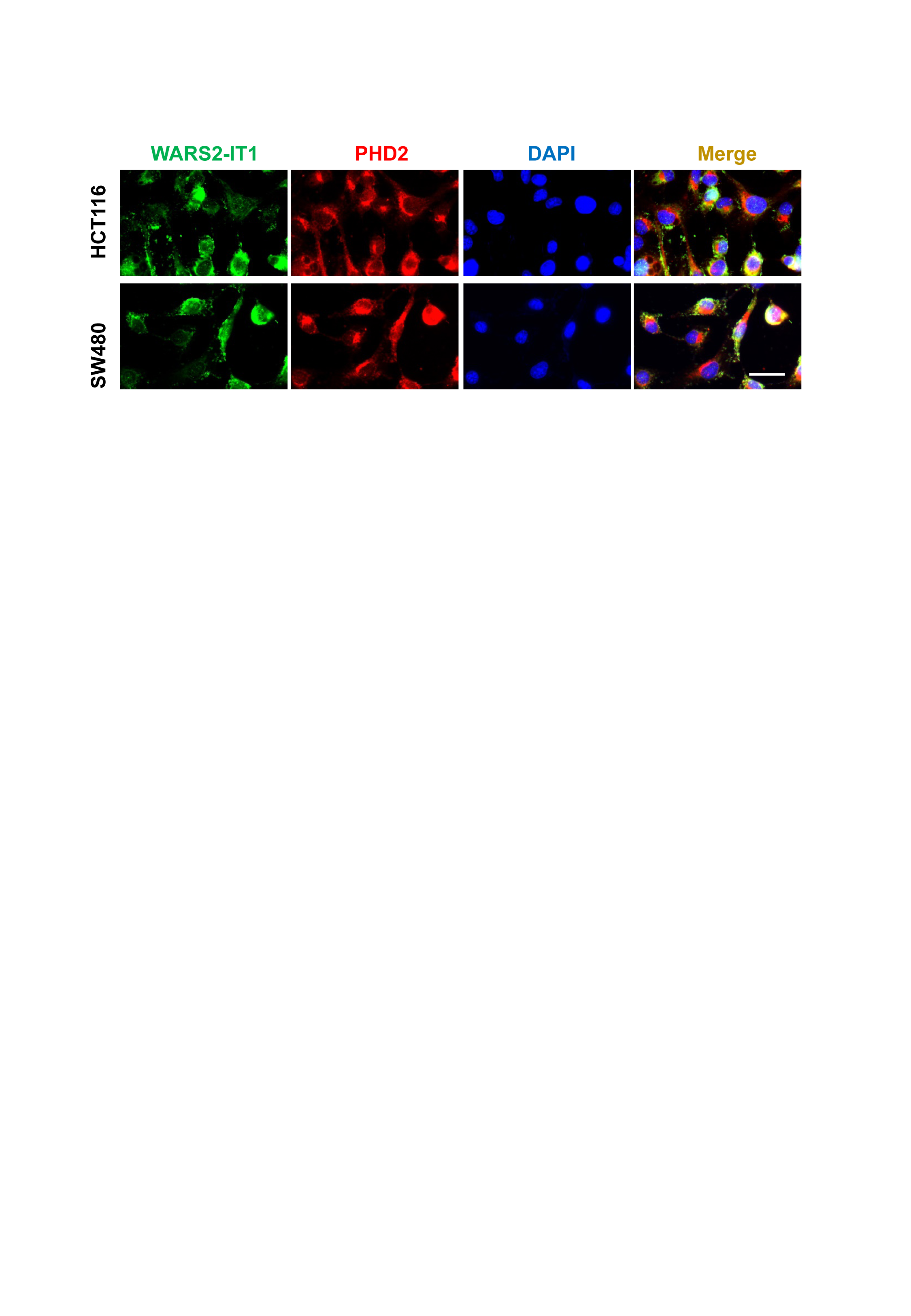

Supplement: Supplementary file 6 — Figure S5 [file 41419_2025_8058_MOESM6_ESM.tif]

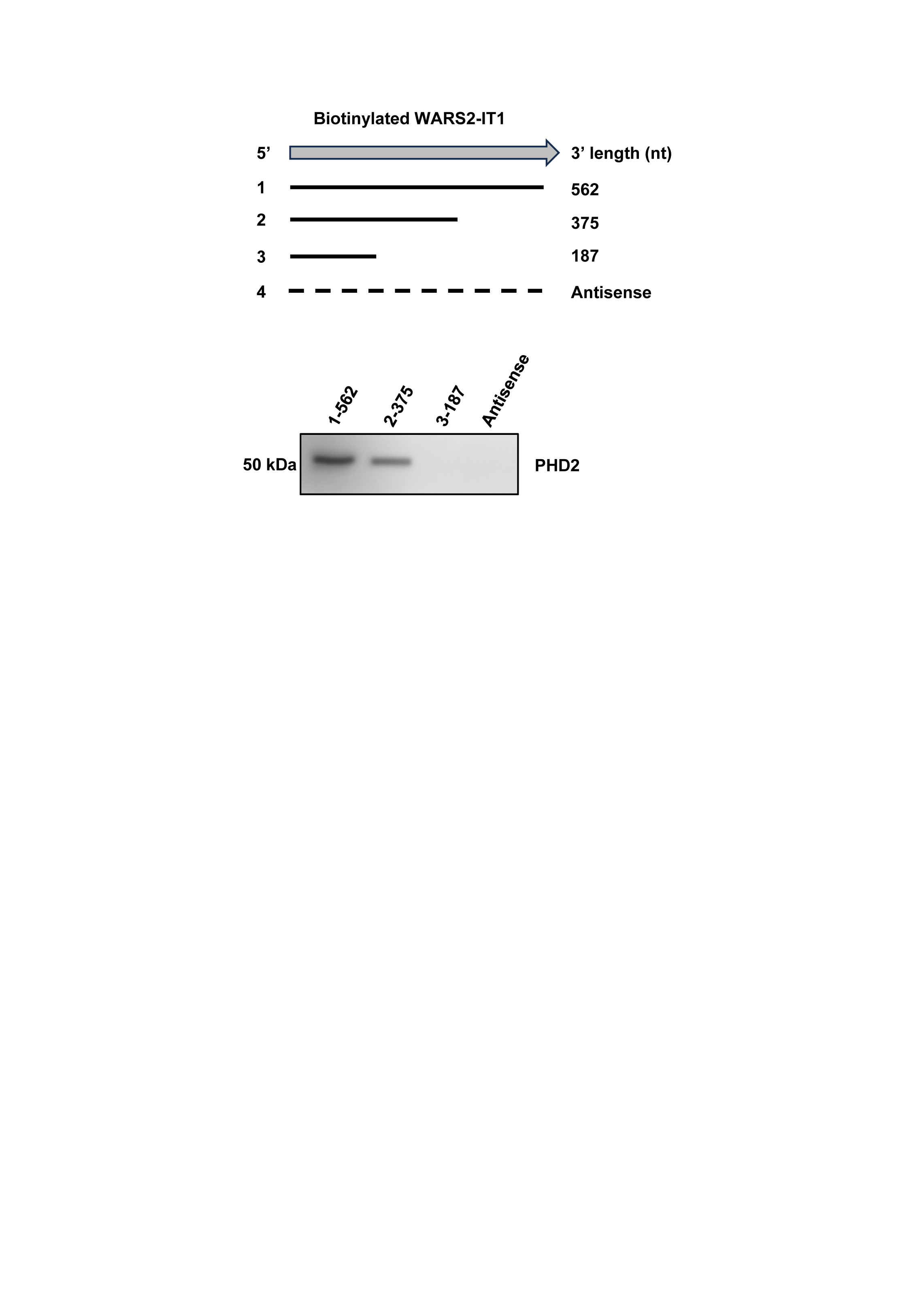

Supplement: Supplementary file 7 — Figure S6 [file 41419_2025_8058_MOESM7_ESM.tif]

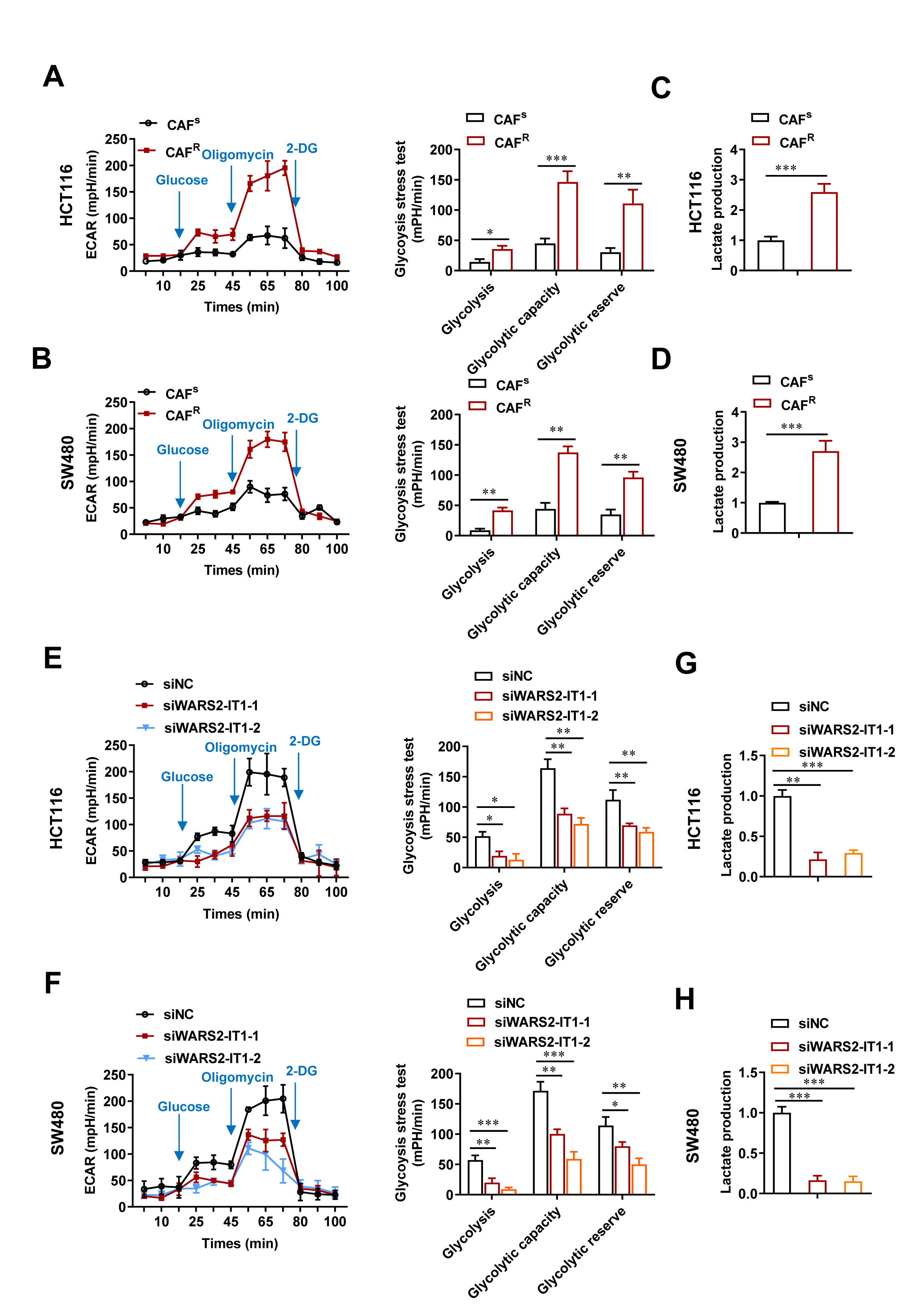

Supplement: Supplementary file 8 — Figure S7 [file 41419_2025_8058_MOESM8_ESM.tif]

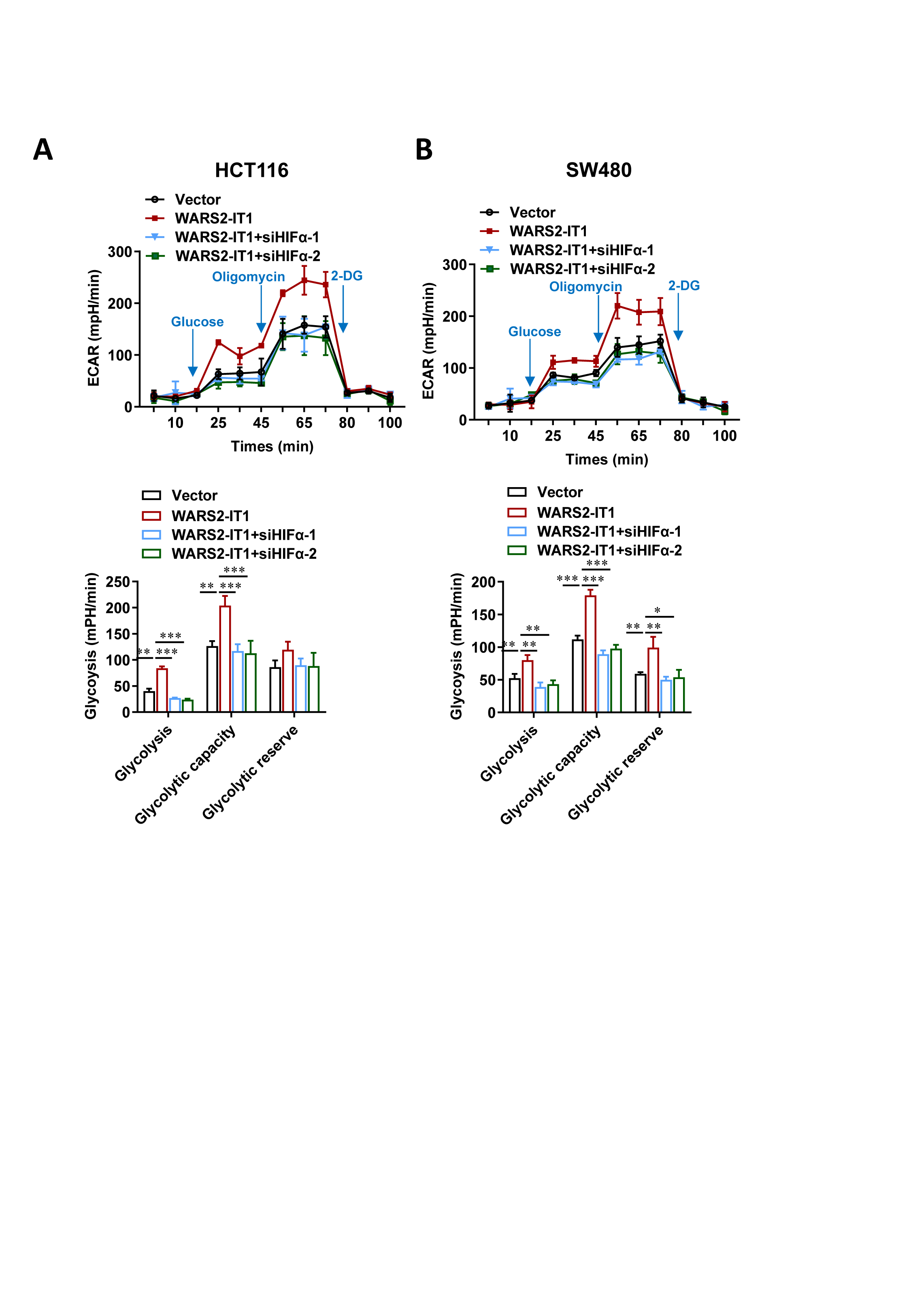

Supplement: Supplementary file 9 — Figure S8 [file 41419_2025_8058_MOESM9_ESM.tif]
