# Supplementary material for: Cancer-associated fibroblast-induced lncRNA WARS2-IT1 confers radioresistance of colorectal cancer via enhancing HIF-1α stability
Source: Cell Death Dis. 2025 Nov 10;16(1):823. doi: 10.1038/s41419-025-08058-1 (PMC12603266; doi:10.1038/s41419-025-08058-1)
Supplement: Supplementary file 10 — Table S1 [file 41419_2025_8058_MOESM10_ESM.docx]

**Table S1**

| **Primer sequences for qRT-PCR (F, forward primer; R, reverse primer)** | | |
| --- | --- | --- |
| WARS2-IT1 | F: GATACGCAAAAAGCTACCACTATG | R: TGAATGCTTTGCTGCTTAGAAATTTCTTC |
| GAPDH | F: CCTTCCGTGTCCCCACT | R: GCCTGCTTCACCACCTTC |
| **Probe for FISH assay** | | |
| WARS2-IT1 | CAGTTCCAAAGTCACTTCCACATTTTCAGGTATCTTTTC | |
| **Sequecnes for ChIP (F, forward primer; R, reverse primer)** | | |
| WARS2-IT1 | F: GTCGCCCAGGCTGCAGTG R: CACAGTTGATGAAGGGCTTGTAATCCCC | |
| **Sequecnes for siRNAs** | | |
| siHIF-1α-1 | CCUAUAUCCC AAUGGAUGAUGTT | |
| siHIF-1α-2 | TTGGAUAUAGGGUUACCUACUAC | |
| siSMAD3-1 | GUGAGCAGAACAGGUAGUAUUAC | |
| siSMAD3-2 | GAGCCUGGUCAAGAAACUCAA | |
| siSMAD2-1 | CAAGTACTCCTTGCTGGATTG | |
| siSMAD2-2 | CATGATCCAGTATCACAGTAT | |
| si-WARS2-IT1-1 | ACAUAGUGGUAGCUUUUUGCG | |
| si-WARS2-IT1-2 | AGUUUCAUUCCAUAUGUCCAA |  |
