# Supplementary material for: Cancer-associated fibroblast-induced lncRNA WARS2-IT1 confers radioresistance of colorectal cancer via enhancing HIF-1α stability
Source: Cell Death Dis. 2025 Nov 10;16(1):823. doi: 10.1038/s41419-025-08058-1 (PMC12603266; doi:10.1038/s41419-025-08058-1)
Supplement: Supplementary file 12 — Full and uncropped western blots [file 41419_2025_8058_MOESM12_ESM.pptx]

## Slide 1
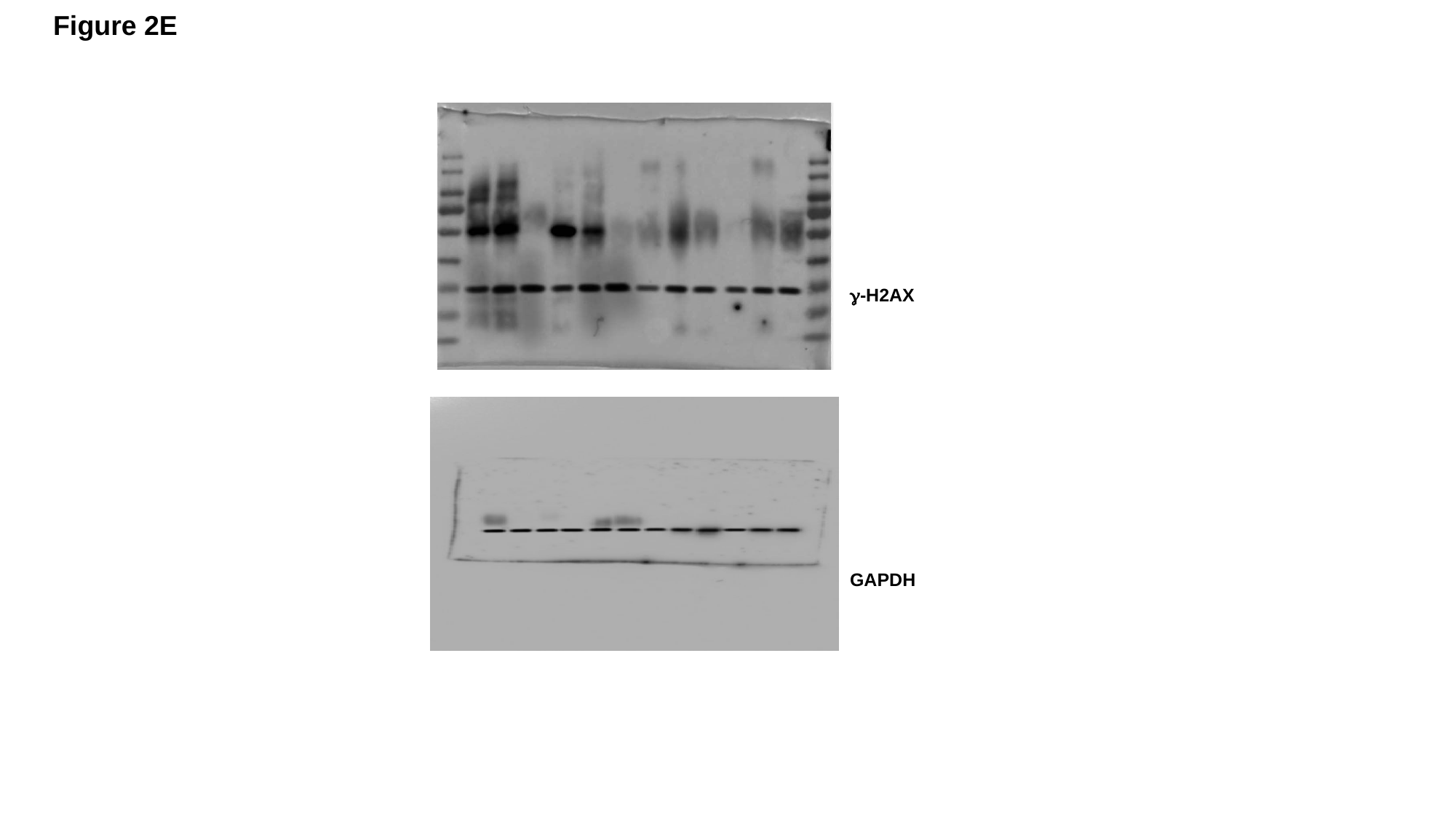

Figure 2E
g-H2AX
GAPDH

## Slide 2
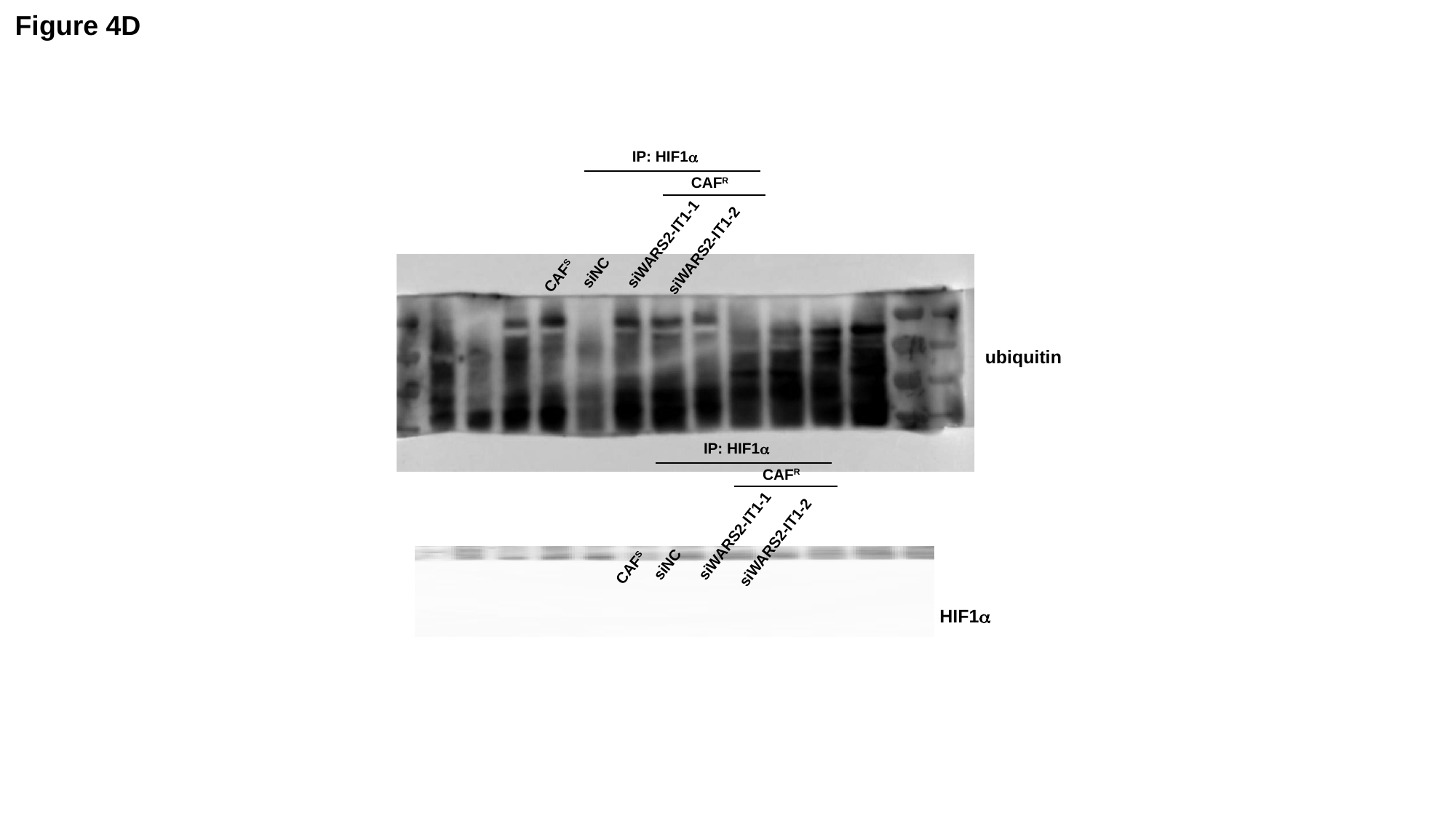

Figure 4D
IP: HIF1a
CAFR
siWARS2-IT1-1
siWARS2-IT1-2
siNC
CAFS
ubiquitin
IP: HIF1a
CAFR
siWARS2-IT1-1
siWARS2-IT1-2
siNC
CAFS
HIF1a

## Slide 3
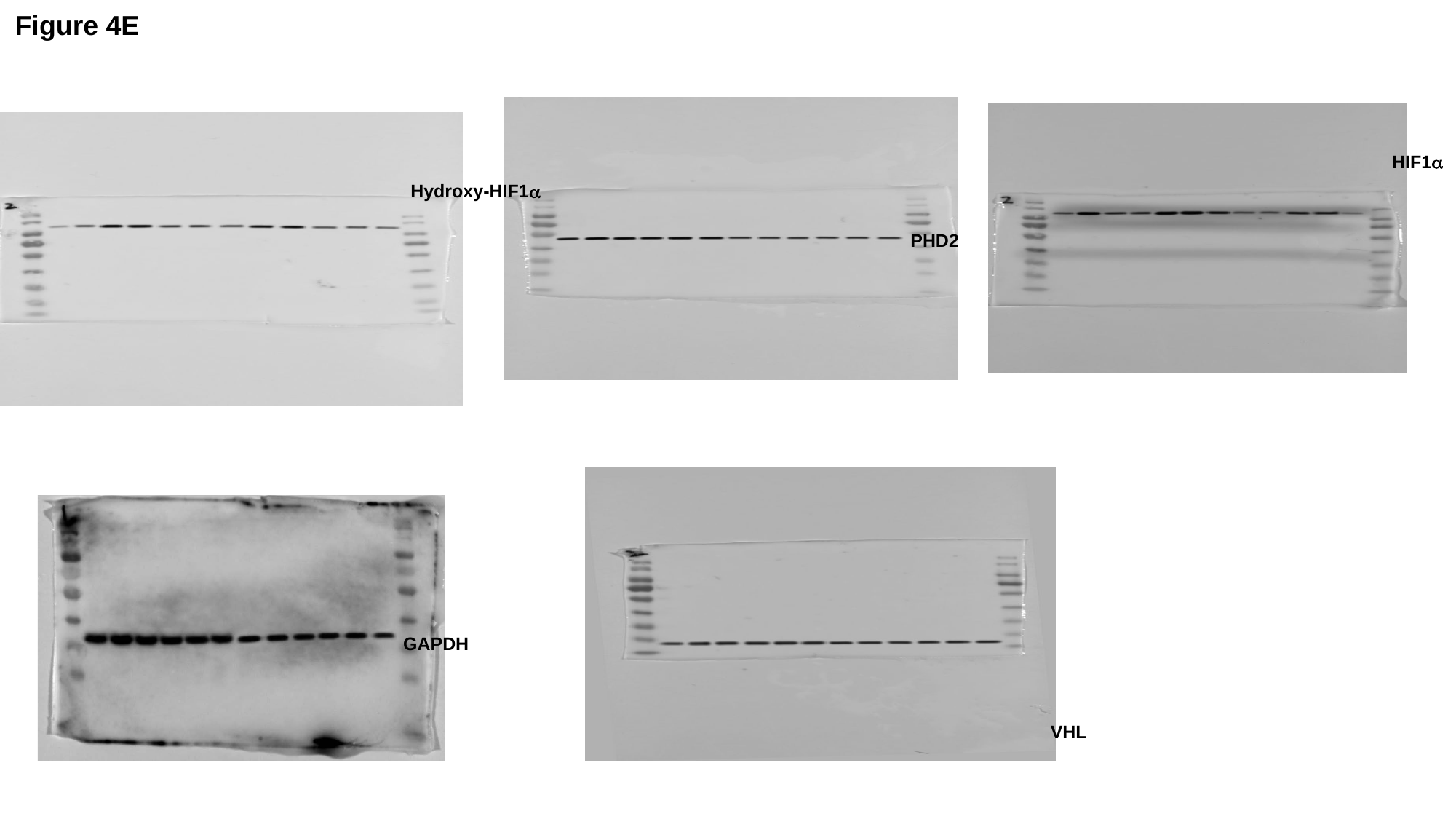

Figure 4E
HIF1a
Hydroxy-HIF1a
PHD2
GAPDH
VHL

## Slide 4
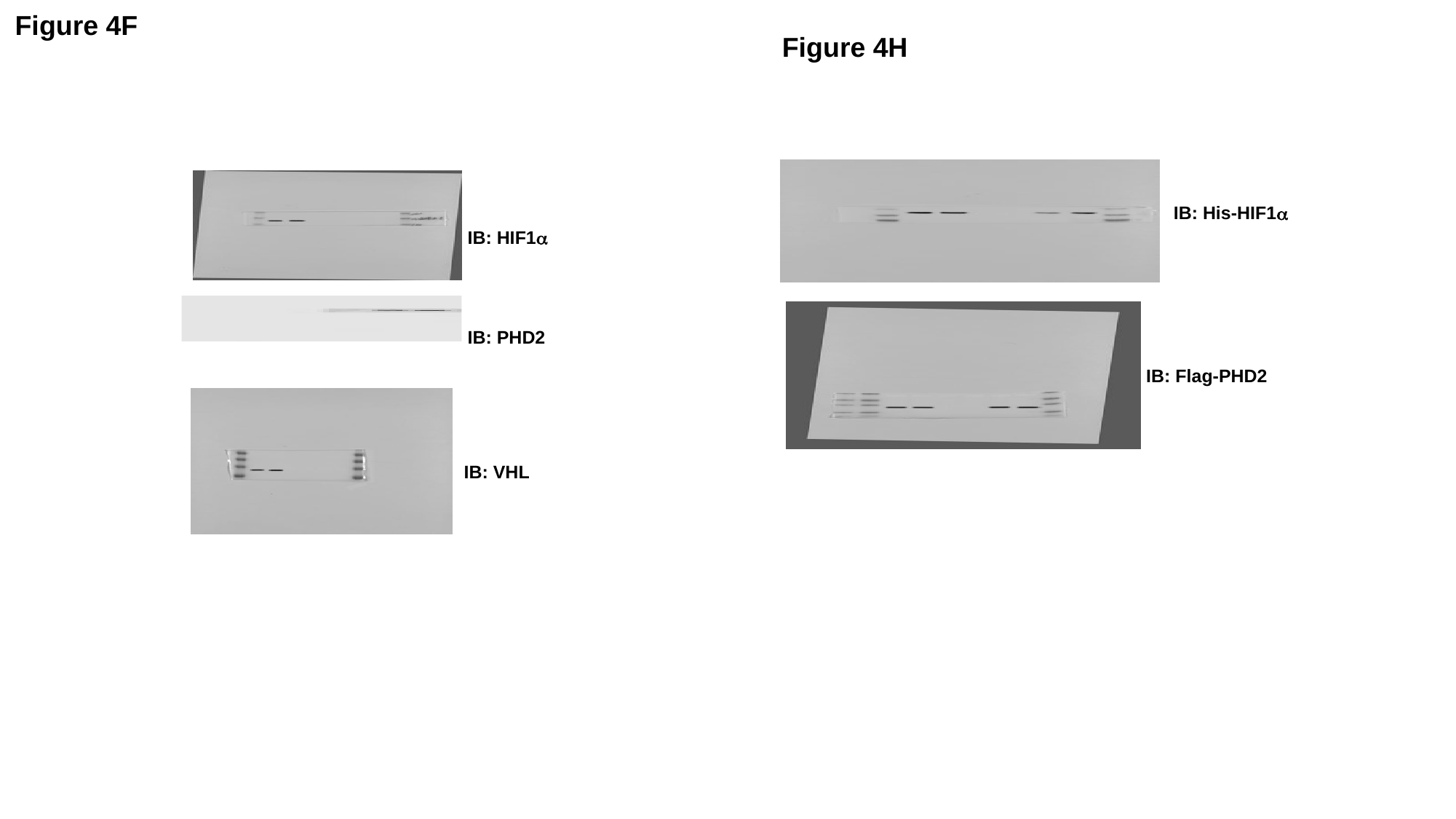

Figure 4F
Figure 4H
IB: His-HIF1a
IB: HIF1a
IB: PHD2
IB: Flag-PHD2
IB: VHL

## Slide 5
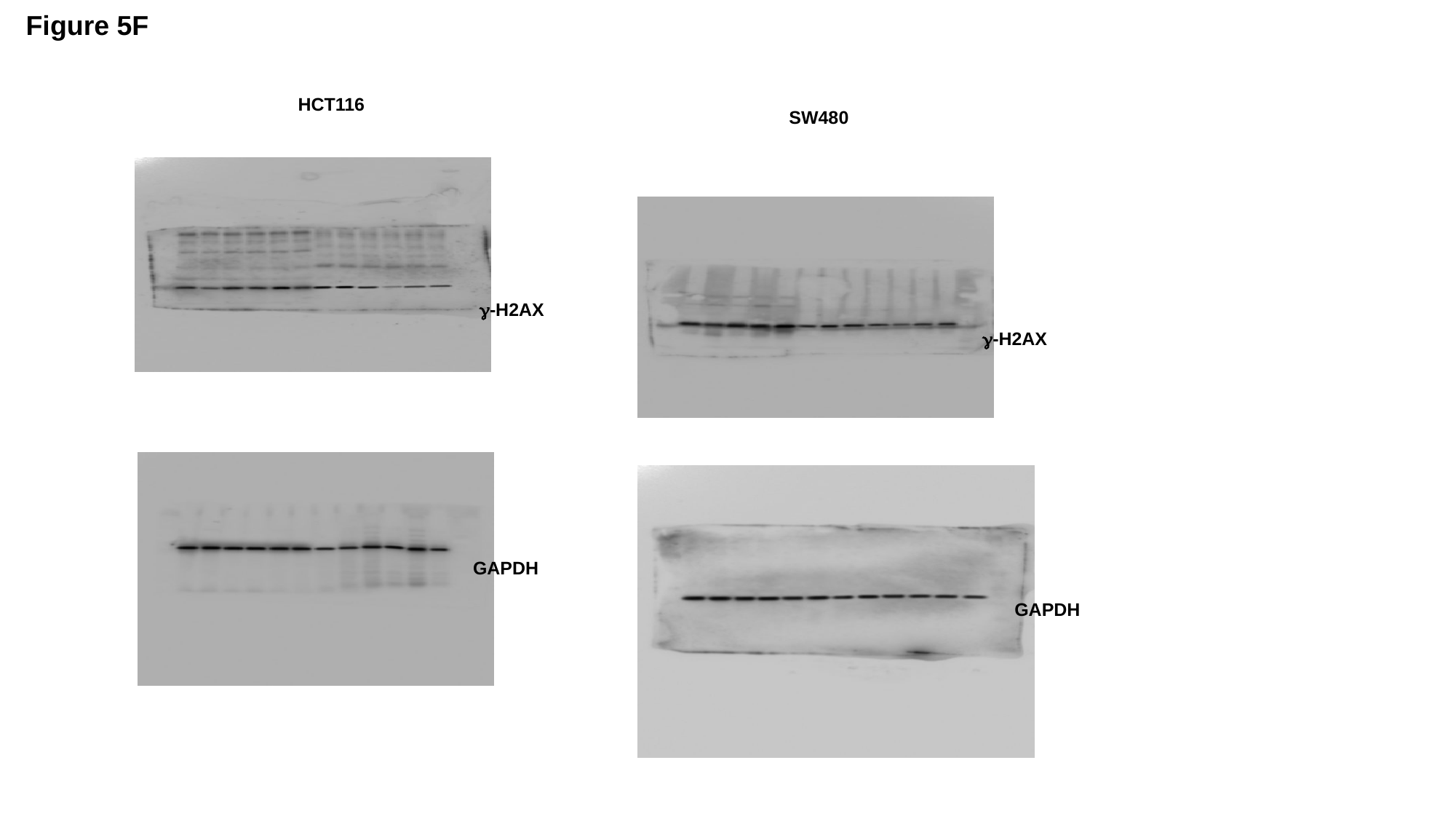

Figure 5F
HCT116
SW480
g-H2AX
g-H2AX
GAPDH
GAPDH

## Slide 6
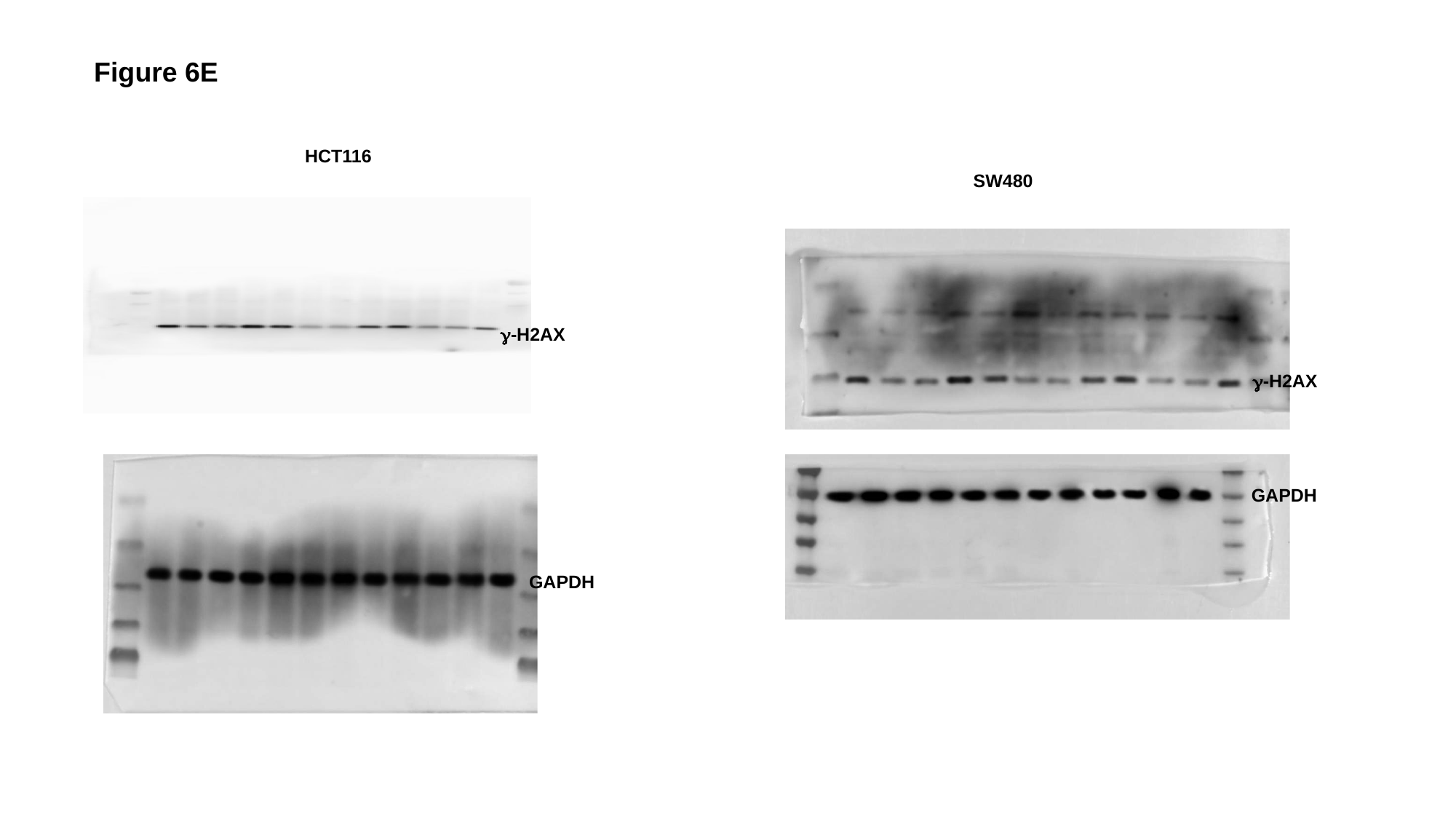

Figure 6E
HCT116
SW480
g-H2AX
g-H2AX
GAPDH
GAPDH

## Slide 7
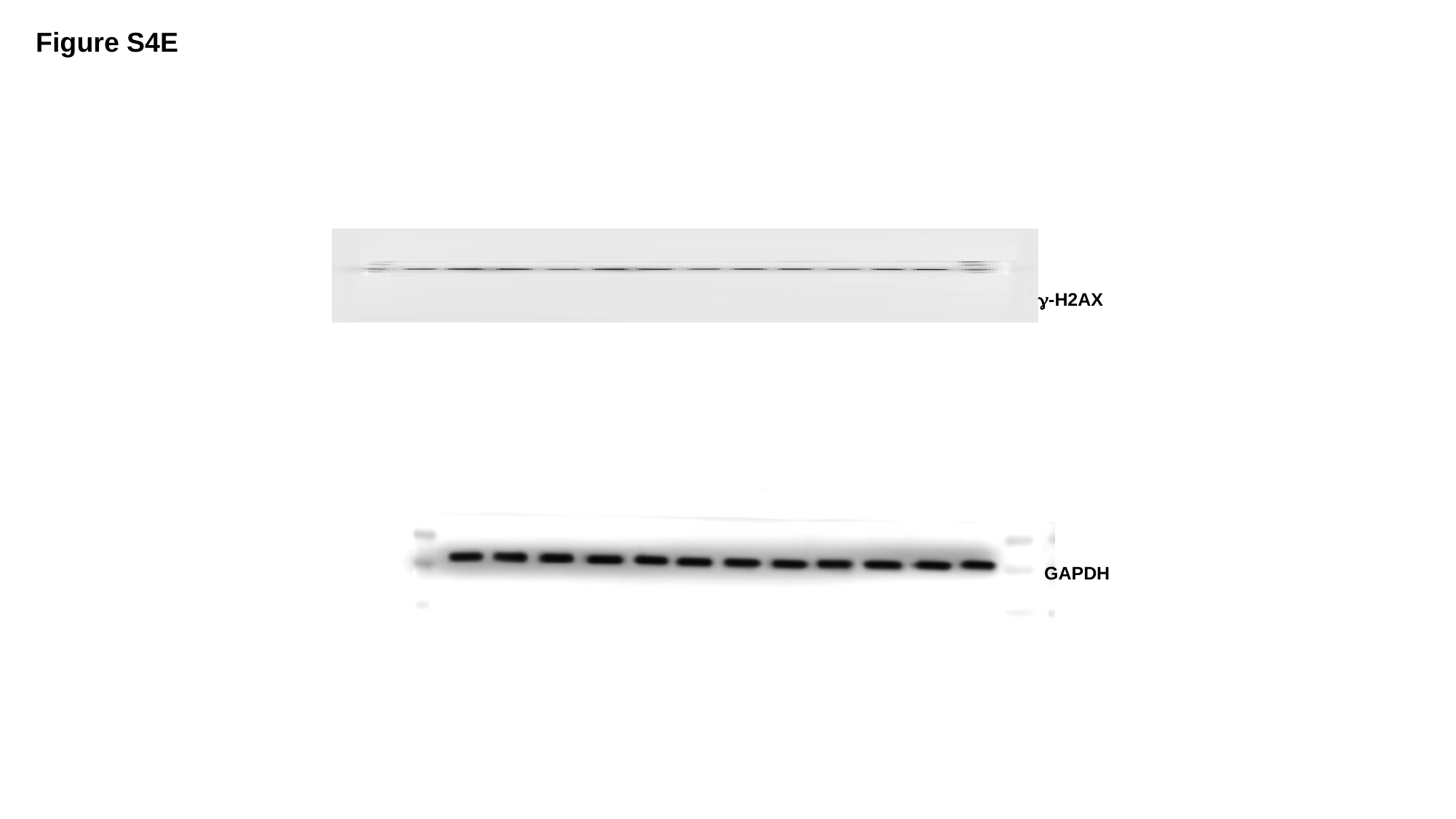

Figure S4E
g-H2AX
GAPDH

## Slide 8
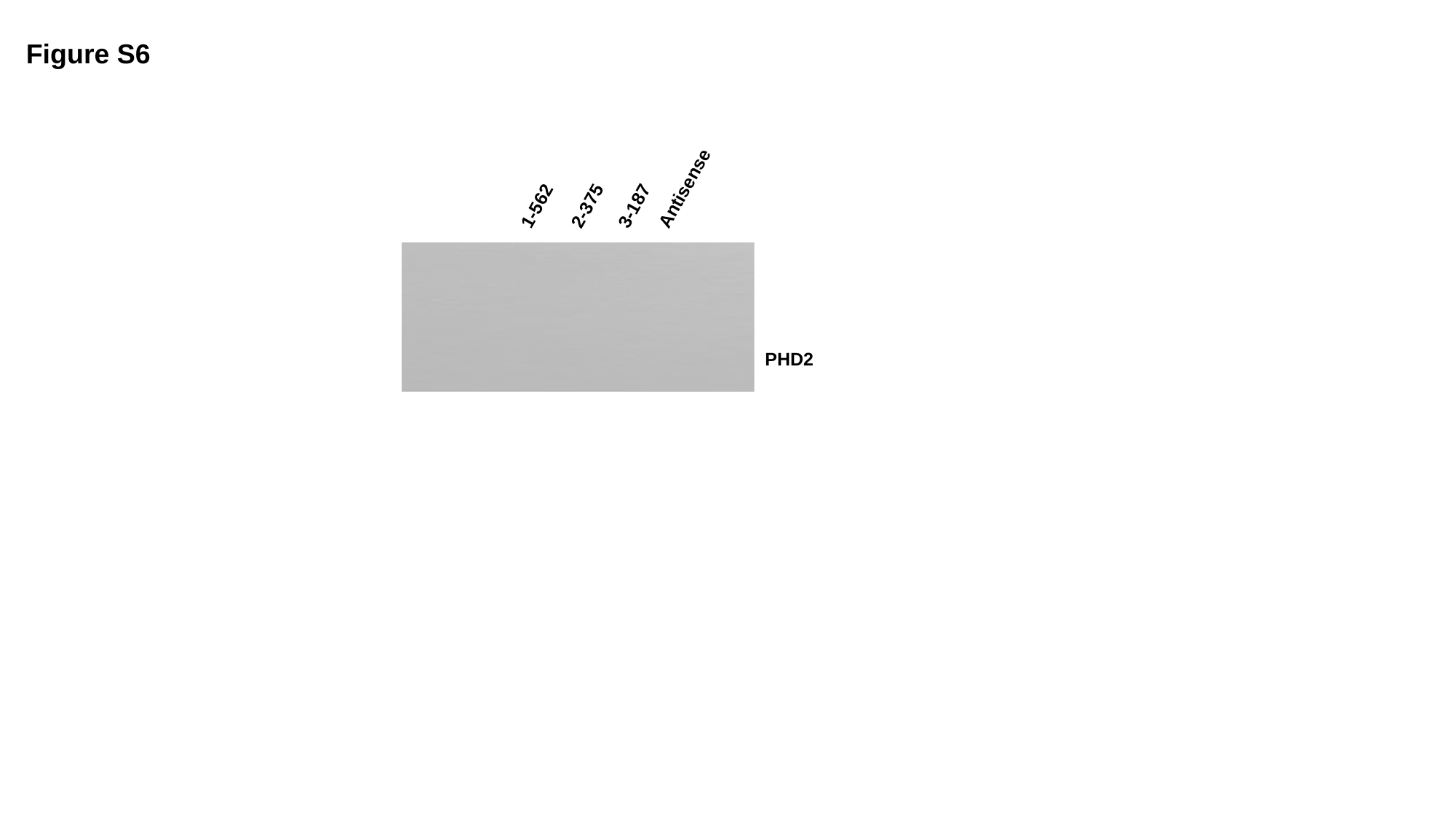

Figure S6
Antisense
1-562
2-375
3-187
PHD2
